# A harmonized global gridded transpiration product based on collocation analysis

**DOI:** 10.1038/s41597-024-03425-7

**Published:** 2024-06-07

**Authors:** Changming Li, Juntai Han, Ziwei Liu, Zhuoyi Tu, Hanbo Yang

**Affiliations:** State Key Laboratory of Hydroscience and Engineering, Department of Hydraulic Engineering, Tsinghua University, Beijing, 100084 China

**Keywords:** Hydrology, Hydrology

## Abstract

Transpiration (T) is pivotal in the global water cycle, responding to soil moisture, atmospheric stress, climate changes, and human impacts. Therefore, establishing a reliable global transpiration dataset is essential. Collocation analysis methods have been proven effective for assessing the errors in these products, which can subsequently be used for multisource fusion. However, previous results did not consider error cross-correlation, rendering the results less reliable. In this study, we employ collocation analysis, taking error cross-correlation into account, to effectively analyze the errors in multiple transpiration products and merge them to obtain a more reliable dataset. The results demonstrate its superior reliability. The outcome is a long-term daily global transpiration dataset at 0.1°from 2000 to 2020. Using the transpiration after partitioning at FLUXNET sites as a reference, we compare the performance of the merged product with inputs. The merged dataset performs well across various vegetation types and is validated against *in-situ* observations. Incorporating non-zero ECC considerations represents a significant theoretical and proven enhancement over previous methodologies that neglected such conditions, highlighting its reliability in enhancing our understanding of transpiration dynamics in a changing world.

## Background & Summary

Transpiration (T) comprises approximately 60% of terrestrial evapotranspiration (ET), playing a pivotal role in Water and energy cycles^1,2^. Observational records indicate a substantial warming trend over the past several decades, inducing an evident shift in soil water availability, atmospheric water stress, climate variations, and human influences, which is expected to alter transpiration^3–6^. Nevertheless, quantifying T at regional and larger scales remains an intricate challenge due to heterogeneities in the physical and physiological attributes governing plant water uptake and ecosystem water utilization^7,8^. These challenges have resulted in limited data availability and substantial uncertainties in ecosystem T estimates, further propagating uncertainties in biosphere-atmosphere feedbacks relevant to climate change projections by Earth System models^9,10^.

Over the past decades, multiple models have emerged for estimating global T and ET^11–13^. However, previous studies investigated uncertainties often exceeding two to three times those of total ET in these products^14–16^. Notably, substantial disparities exist among previous studies estimating global T/ET ratios, ranging from 24% to 76% based on satellite observations^14^, 31% to 64% using hydrological models^2^, and 25% to 90% derived from climate models^17^. The improvement of these models is impeded by the lack of suitable datasets for direct T product validation, mechanism testing, and parameter constraints^18^. Validation efforts are often hindered by sparse *in situ* data^10^ and the limited availability of measurement techniques and datasets at the requisite spatial and temporal scales^16,19^.

Collocation methods have recently emerged as promising techniques for estimating random error variances and data-truth correlations in collocated inputs^15,20–22^. These methods do not demand a high-quality reference dataset but instead rely on the availability of spatially and temporally corresponding datasets^23,24^. Collocation methods have found widespread application in assessing various geophysical variables, encompassing soil moisture^25,26^, precipitation^27,28^, ocean wind speed^29,30^, leaf area index^31^, total water storage^32^ sea ice thickness and surface salinity^33^, and near-surface air temperature^34^.

Recent efforts have applied collocation analysis to assess transpiration estimates. Bright *et al*.^35^ utilized the additive triple collocation (TC) model to scrutinize the performance of diverse models in estimating daily transpiration. Park *et al*.^15^ amalgamated three products (e.g., ERA5L, GLDAS, and MERRA2) using TC-derived error information over East Asia. Li *et al*.^36^ utilized the extended double instrumental variable (EIVD) method to evaluate the uncertainty of three global gridded transpiration datasets (e.g., ERA5L, GLDAS, and GLEAM). The findings of these studies corroborated the reliability of the collocation method and highlighted its increased suitability for assessing transpiration estimates.

The mathematical premise of collocation analysis assumes that multiple products are mutually independent^20^, meaning that the random errors of these products are not correlated. If this error cross correlation (ECC) assumption is not met, zero it can lead to significant errors in the results. However, in practice, many products use the same data source for driving or calibration, making it challenging to satisfy the zero ECC assumption. For example, Li *et al*.^36^ ‘s global T products evaluation research revealed clear non-zero ECC conditions between ERA5L, GLDAS, and GLEAM. The subsequent developments in collocation analysis, such as extended collocation (EC) or EIVD methods, have introduced specific approaches for calculating ECC, thereby relaxing this assumption to some extent.

Recent studies have demonstrated that the framework of collocation analysis can be employed for the analysis and fusion of transpiration products. However, they did not account for the potential existence of ECC, which is disadvantageous for multisource data fusion. Unlike other violations of mathematical assumptions adopted by TC, they cannot be effectively mitigated through rescaling or compensated for by equal magnitude adjustments across inputs. Thus, the implications of non-zero ECC in the context of merging strategies are a critical consideration often overlooked in previous research. This oversight can lead to significant biases and inaccuracies. We aim to bridge this gap by systematically accounting for non-zero ECC in weight calculation, contributing to a more robust and accurate assessment.

In summary, this study addresses the challenges posed by the difficulty in estimating global transpiration and the limited assessment of existing products. We intend to employ a collocation analysis approach considering non-zero ECC to analyse the errors in four commonly used transpiration products. Subsequently, we will perform multisource data fusion to obtain more reliable and robust global gridded transpiration data. These data will be compared with input datasets and other fusion methods at both site and global scales to assess the robustness of the fusion results. This research will provide the scientific community with reliable data support for analysing the spatiotemporal variability trends and underlying reasons for transpiration under changing environmental conditions. The primary findings of this investigation include:

1. The collocation analysis method can effectively be used for error analysis of global-scale transpiration products. The calculated random error variances can be used for further data fusion when considering the correlation of random errors in the products.
2. At the site scale, compared to the mean transpiration estimated by three commonly used ET partition methods as a reference, the fused product shows improved accuracy, especially a significant reduction in errors compared to the results of simple averaging and traditional TC fusion without considering non-zero ECC.
3. The fused results perform better than other products for different Plant Functional Types (PFTs). In some sites, PMLv2 exhibits superior performance, partly validated by its use of FLUXNET site data for calibration, supporting the reliability of the fused results.
4. Significant differences emerged among different products when examining the global multiyear annual transpiration and trend. Site-scale assessment validate the improved performance of the merged product.
5. When utilizing the error information derived from collocation analysis for merging, it is crucial to consider the potential presence of non-zero ECC. Comparing the merging schemes with and without considering non-zero ECC, it was found that considering ECC improves the accuracy of the merging process. Additionally, when using collocation analysis, it is necessary to identify which products may have ECC in advance, providing more effective support for data merging and obtaining more accurate product error information.

Our collocation-based data merging approach demonstrated promising potential for merging transpiration products. The resulting product exhibited good overall performance and met the requirements for more detailed research. For future enhancement, further evaluation in specific regions will be necessary to identify potential strengths and weaknesses of the product. Moreover, dynamic weights could be computed by considering suitable merging periods for different products to enhance the quality of the merged product, and more sophisticated combination schemes could be explored to improve accuracy.

## Methods

In this study, the fusion of products consisted of three steps: (1) the extended double instrumental variable technique (EIVD) was used to calculate the random error variance of the selected input products; (2) aiming for minimum MSE, the weights of different products on each grid were calculated considering non-zero error-cross-correlation (ECC); (3) the products were fused according to the weights to obtain a long sequence. The detailed derivation processes of errors and weights could be found in the supplementary. In addition, to evaluate the performance of the fusion results, we compared the results to three independent methods for partitioning T from ET. As a benchmark, we calculated the means of these three methods to obtain site-scale T data for the long time series. Six statistical metrices are used for evaluation, including the root-mean-squared error (RMSE), unbiased RMSE (ubRMSE), mean absolute error (MAE), relative bias (RB), modified Kling-Gupta efficiency^37^ (KGE) and Pearson correlation coefficient (R).

### Collocation analysis

#### Extended instrumental variable technique

The EIVD method^38^ used in this study is a type of collocation analysis that combines the traditional triple collocation (TC) method^20^ with the extended collocation (EC) method^39^, which considers non-zero ECC. Therefore, we must first introduce the TC method to derive the rationale behind the EIVD method.

The commonly used error structure for triple collocation analysis (TCA) is:

1 $$i={\alpha }_{i}+{\beta }_{i}\Theta +{\varepsilon }_{i}$$

where *I* ∈ [*X, Y, Z*] are three spatially and temporally collocated data sets; Θ is the unknown true signal for relative geographical variable; *α*_*i*_ and *β*_*i*_ are additive and multiplicative bias factors against the true signal, respectively; *ε*_*i*_ is the additive zero-mean random error.

The basic assumptions adopted in TC are as follows: (i) Linearity between true signal and data sets, (ii) signal and error stationarity, (iii) independency between random error and true signal (error orthogonality), (iv) independence between random errors (zero error cross-correlation, zero ECC). Although many studies have indicated that some of these assumptions are often violated in practice^21,28,40^, the formulation based on these assumptions is still the most robust implementation^41^.

The data sets first need to be rescaled against an arbitrary reference (e.g., *X*). The others are scaled through a TC-based rescaling scheme:

2 $${Y}^{X}={\beta }_{Y}^{X}\left(Y-\bar{Y}\right)+\bar{X}\,{Z}^{X}={\beta }_{Z}^{X}\left(Z-\bar{Z}\right)+\bar{X}$$

The overbar denotes the mean value, and ${\beta }_{Y}^{X}$ and ${\beta }_{Z}^{X}$ are the scaling factors as:

3 $$\left\{\begin{array}{c}{\beta }_{Y}^{X}=\frac{{\beta }_{X}}{{\beta }_{Y}}=\frac{\left\langle \left(X-\bar{X}\right)\left(Z-\bar{Z}\right)\right\rangle }{\left\langle \left(Y-\bar{Y}\right)\left(Z-\bar{Z}\right)\right\rangle }=\frac{{\sigma }_{XZ}}{{\sigma }_{YZ}}\\ {\beta }_{Z}^{X}=\left(\frac{{\beta }_{X}}{{\beta }_{Z}}\right)=\frac{\left\langle \left(X-\bar{X}\right)\left(Y-\bar{Y}\right)\right\rangle }{\left\langle \left(Z-\bar{Z}\right)\left(Y-\bar{Y}\right)\right\rangle }=\frac{{\sigma }_{XY}}{{\sigma }_{ZY}}\end{array}\right.$$

where <∙> is the average operator, *σ*_*ij*_ is the covariance of data sets *i* and *j*.

Subsequently, the error variances could be estimated by averaging the cross-multiplied data set differences as follows:

4 $$\left\{\begin{array}{c}{\sigma }_{{\varepsilon }_{X}}^{2}=\left\langle \left(X-{Y}^{X}\right)\left(X-{Z}^{X}\right)\right\rangle \\ {\sigma }_{{\varepsilon }_{Y}^{X}}^{2}={\beta }_{Y}^{X2}{\sigma }_{{\varepsilon }_{Y}}^{2}=\left\langle \left({Y}^{X}-X\right)\left({Y}^{X}-{Z}^{X}\right)\right\rangle \\ {\sigma }_{{\varepsilon }_{Z}^{X}}^{2}={\beta }_{Z}^{X2}{\sigma }_{{\varepsilon }_{Z}}^{2}=\left\langle \left({Z}^{X}-X\right)\left({Z}^{Y}-{Y}^{X}\right)\right\rangle \end{array}\right.$$

Expanding the bracket and expressing the rescaling factors yields:

5 $$\left\{\begin{array}{c}{\sigma }_{{\varepsilon }_{X}}^{2}={\sigma }_{X}^{2}-\frac{{\sigma }_{XY}{\sigma }_{XZ}}{{\sigma }_{YZ}}\\ {\sigma }_{{\varepsilon }_{Y}}^{2}={\sigma }_{Y}^{2}-\frac{{\sigma }_{YX}{\sigma }_{YZ}}{{\sigma }_{XZ}}\\ {\sigma }_{{\varepsilon }_{Z}}^{2}={\sigma }_{Z}^{2}-\frac{{\sigma }_{ZX}{\sigma }_{ZY}}{{\sigma }_{XY}}\end{array}\right.$$

Following the classic TC analysis, the problem is generalized for an arbitrary number of *N* data sets (Zwieback *et al*., 2012) by relaxing the zero ECC assumption for specific data sets combination. Here, we use a quadruple input $\left[i,j,k,l\,{\rm{with}}\,{\sigma }_{{\varepsilon }_{i}{\varepsilon }_{j}}\ne 0\right]$ for expression. The data set variances and covariances write as:

6 $${\sigma }_{ij}=\left\{\begin{array}{c}{\beta }_{i}{\beta }_{j}{\sigma }_{\Theta }^{2}\\ {\beta }_{i}{\beta }_{j}{\sigma }_{\Theta }^{2}+{\sigma }_{{\varepsilon }_{i}{\varepsilon }_{j}}\end{array}\begin{array}{c}\forall i,j\,{\rm{with}}\,{\sigma }_{{\varepsilon }_{i}{\varepsilon }_{j}}=0\\ \forall i,j\,{\rm{with}}\,{\sigma }_{{\varepsilon }_{i}{\varepsilon }_{j}}\ne 0\end{array}\right.$$

The sensitivity and absolute error variance of the data set follow:

7 $${\beta }_{j}^{2}{\sigma }_{\Theta }^{2}=\frac{{\sigma }_{jk}{\sigma }_{jl}}{{\sigma }_{kl}}\,{\sigma }_{{\varepsilon }_{j}}^{2}={\sigma }_{j}^{2}-\frac{{\sigma }_{jk}{\sigma }_{jl}}{{\sigma }_{kl}}$$

The cross-multiplied factors can be estimated by:

8 $${\beta }_{i}{\beta }_{j}{\sigma }_{\Theta }^{2}=\frac{{\sigma }_{ik}{\sigma }_{jl}}{{\sigma }_{kl}}\,{\sigma }_{{\varepsilon }_{i}{\varepsilon }_{j}}={\sigma }_{ij}-\frac{{\sigma }_{ik}{\sigma }_{jl}}{{\sigma }_{kl}}$$

The above equations could be expressed in matrix notation with **y** = **Ax** as:

9 $${\bf{y}}=\left(\begin{array}{c}{\sigma }_{i}^{2}\\ {\sigma }_{ij}\\ \frac{{\sigma }_{jl}{\sigma }_{jk}}{{\sigma }_{lk}}\\ \frac{{\sigma }_{ij}{\sigma }_{kl}}{{\sigma }_{jl}}\end{array}\right){\bf{A}}=\left(\begin{array}{cccc}1 & 0 & 1 & 0\\ 0 & 1 & 0 & 1\\ 1 & 0 & 0 & 0\\ 0 & 1 & 0 & 0\end{array}\right){\bf{x}}=\left(\begin{array}{c}{\beta }_{i}^{2}{\sigma }_{\Theta }^{2}\\ {\beta }_{i}{\beta }_{j}{\sigma }_{\Theta }^{2}\\ {\sigma }_{{\varepsilon }_{j}}^{2}\\ {\sigma }_{{\varepsilon }_{i}{\varepsilon }_{j}}\end{array}\right)$$

Where **y** is the known observations vector, **A** is the design matrix, **x** is the unknown parameters vector. The least-squared solution for unknown **x** is then solved by:

10 $${\bf{x}}={\left({{\bf{A}}}^{{\bf{T}}}{\bf{A}}\right)}^{-1}{{\bf{A}}}^{{\bf{T}}}{\bf{y}}$$

The matrix **(A**^**T**^**A)** must have full rank (invertible) to guarantee that the collocation system is solvable. For any number of *N* > 3, this requirement could be expressed as Each data set must be a member of at least one triplet with mutually zero ECC (Gruber *et al*., 2016a). For example, input with five data sets [*a, b, c, d, e*] can assume at most 2 sets with 2 non-zero ECC pairs (like ${\sigma }_{{\varepsilon }_{a}{\varepsilon }_{b}}\& {\sigma }_{{\varepsilon }_{a}{\varepsilon }_{c}}\ne 0$ and ${\sigma }_{{\varepsilon }_{a}{\varepsilon }_{b}}\& {\sigma }_{{\varepsilon }_{c}{\varepsilon }_{d}}\ne 0$) or ${{\rm{C}}}_{5}^{2}=10$ sets with 1 non-zero ECC pair, etc.

The instrumental variable algorithm introduces a temporally lag-1 [day] series of the select product (e.g., ${X}_{t-1}={\alpha }_{X}+{\beta }_{X}{\Theta }_{t-1}+{\varepsilon }_{X,t-1}$) as the third input for TC (Su *et al*., 2014). Such process includes another assumption that all data sets contain serially white errors (i.e., $\langle {\varepsilon }_{i,t}{\varepsilon }_{i,t-1}\rangle =0$, zero auto-correlation). Furthermore, by adopting the designed matrix in extended collocation (EC)^39^, Dong *et al*.^42^ present the extended double instrumental variable technique (denoted as EIVD) to estimate the error variance matrix with only two independent data sets.

For a triplet input $\left[i,j,k\,{\rm{with}}\,{\sigma }_{{\varepsilon }_{i}{\varepsilon }_{j}}\ne 0\right]$. The dynamic range ratio scaling factors can be estimated as follows:

11 $${s}_{ij}\equiv \frac{{\beta }_{i}}{{\beta }_{j}}=\sqrt{\frac{{L}_{ii}}{{L}_{jj}}}$$

where *L*_*ii*_ = ⟨*i*_*t*_ *i*_*t*-1_⟩ is the auto-covariance of inputs. Subsequently, the sensitivity and absolute error variance of the data set follow:

12 $${\beta }_{j}^{2}{\sigma }_{\Theta }^{2}={\sigma }_{ij}\sqrt{\frac{{L}_{ii}}{{L}_{jj}}}\,{\sigma }_{{\varepsilon }_{j}}^{2}={\sigma }_{ij}\sqrt{\frac{{L}_{ii}}{{L}_{jj}}}-{\sigma }_{i}^{2}$$

The cross-multiplied factors can be estimated by:

13 $${\beta }_{i}{\beta }_{j}{\sigma }_{\Theta }^{2}={\sigma }_{ik}\sqrt{\frac{{L}_{jj}}{{L}_{kk}}}={\sigma }_{jk}\sqrt{\frac{{L}_{ii}}{{L}_{kk}}}\,{\sigma }_{{\varepsilon }_{i}{\varepsilon }_{j}}={\sigma }_{ij}-{\beta }_{i}{\beta }_{j}{\sigma }_{\Theta }^{2}$$

Hence, for a triplet with the input of $\left[X,Y,Z\,{\rm{with}}\,{\sigma }_{{\varepsilon }_{X}{\varepsilon }_{Y}}\ne 0\right]$: the matrix notation of the above system with **y** = **Ax** is given as:

14 $${\bf{y\; =}}{\left(\begin{array}{c}{\sigma }_{X}^{2}\\ {\sigma }_{Y}^{2}\\ {\sigma }_{Z}^{2}\\ {\sigma }_{XY}\\ {\sigma }_{XZ}\sqrt{\frac{{L}_{XX}}{{L}_{ZZ}}}\\ {\sigma }_{YZ}\sqrt{\frac{{L}_{YY}}{{L}_{ZZ}}}\\ {\sigma }_{ZX}\sqrt{\frac{{L}_{ZZ}}{{L}_{XX}}}\\ {\sigma }_{ZY}\sqrt{\frac{{L}_{ZZ}}{{L}_{YY}}}\\ {\sigma }_{XZ}\sqrt{\frac{{L}_{YY}}{{L}_{ZZ}}}\\ {\sigma }_{YZ}\sqrt{\frac{{L}_{XX}}{{L}_{ZZ}}}\end{array}\right)}_{{\bf{10}}\times {\bf{1}}}{\bf{A\; =}}{\left({\left(\begin{array}{cccc} &  & {{\bf{I}}}_{{\bf{4}}\times {\bf{4}}} & \\ 1 & 0 & 0 & 0\\ 0 & 1 & 0 & 0\\ 0 & 0 & 1 & 0\\ 0 & 0 & 1 & 0\\ 0 & 0 & 0 & 1\\ 0 & 0 & 0 & 1\end{array}\right)}_{{\bf{6}}\times {\bf{4}}}\begin{array}{c}{{\bf{I}}}_{{\bf{4}}\times {\bf{4}}}\\ \\ \\ {{\bf{0}}}_{{\bf{6}}\times {\bf{4}}}\\ \\ \\ \end{array}\right)}_{{\bf{10}}\times {\bf{8}}}{\bf{x\; =}}{\left(\begin{array}{c}{\beta }_{X}^{2}{\sigma }_{\Theta }^{2}\\ {\beta }_{Y}^{2}{\sigma }_{\Theta }^{2}\\ {\beta }_{Z}^{2}{\sigma }_{\Theta }^{2}\\ {\beta }_{X}{\beta }_{Y}{\sigma }_{\Theta }^{2}\\ {\sigma }_{\varepsilon X}^{2}\\ {\sigma }_{\varepsilon Y}^{2}\\ {\sigma }_{\varepsilon Z}^{2}\\ {\sigma }_{\varepsilon X\varepsilon Y}\end{array}\right)}_{{\bf{8}}\times {\bf{1}}}$$

Likewise, the least-squared solution for unknown **x** is solved by Eq. (10).

### Weight estimation

Our objective is to predict an uncertain variable, such as transpiration (ET) over time at a specific location, by utilizing parent products that may contain random errors. The underlying concept of weighted averaging is to extract independent information from multiple data sources to enhance prediction accuracy by mitigating the effects of random errors. The effectiveness of this approach relies on the independence of the individual data sources. Weighted averaging has been applied in various fields following the influential work of Bates and Granger^43^, which proposed the optimal combination of forecasts based on a mean square error (MSE) criterion. In this context, the term “optimal” refers to minimizing the variance of residual random errors in the least squares sense. Mathematically, this weighted average can be expressed as follows:

15 $$\bar{x}={\overrightarrow{{\bf{W}}}}^{{\rm{T}}}\overrightarrow{{\bf{X}}}=\mathop{\sum }\limits_{i=1}^{N}{\omega }_{i}{x}_{i}$$

where $\bar{x}$ is the merged estimate; $\overrightarrow{{\bf{X}}}={\left[{x}_{1},\ldots ,{x}_{n}\right]}^{{\rm{T}}}$ contains the temporally collocated estimates from *N* different parent products, which are merged with relative zero-mean random error $\overrightarrow{{\boldsymbol{e}}}={\left[{\varepsilon }_{1},\ldots ,{\varepsilon }_{n}\right]}^{{\rm{T}}}$; and $\overrightarrow{{\bf{W}}}={\left[{\omega }_{1},\ldots ,{\omega }_{n}\right]}^{{\rm{T}}}$ contains the weights assigned to these estimates, where ${\omega }_{i}\in \left[0,1\right]$ and $\sum {\omega }_{i}=1$ ensuring an unbiased prediction.

The averaging weights can be expressed as the solution to the problem:

16 $$\min f\left(\overrightarrow{{\bf{W}}}\right)={\mathbb{E}}{\left({\overrightarrow{{\boldsymbol{e}}}}^{{\rm{T}}}\overrightarrow{{\bf{W}}}\right)}^{2}$$

where ${\mathbb{E}}$() is the operator for mathematical expectation, the solution of this problem is determined by the individual random error characteristics of the input data sets and can be derived from their covariance matrix^43–45^:

17 $$\begin{array}{l}\overrightarrow{{\bf{W}}}={({\overrightarrow{{\bf{I}}}}^{{\rm{T}}}{\mathbb{E}}{({\overrightarrow{{\boldsymbol{e}}}\overrightarrow{{\boldsymbol{e}}}}^{{\rm{T}}})}^{-1}\overrightarrow{{\bf{I}}})}^{-1}{\mathbb{E}}{({\overrightarrow{{\boldsymbol{e}}}\overrightarrow{{\boldsymbol{e}}}}^{{\rm{T}}})}^{-1}\overrightarrow{{\bf{I}}}\\ {\sigma }_{{\varepsilon }_{\bar{x}}}^{2}={({\overrightarrow{{\bf{I}}}}^{{\rm{T}}}{\mathbb{E}}{({\overrightarrow{{\boldsymbol{e}}}\overrightarrow{{\boldsymbol{e}}}}^{{\rm{T}}})}^{-1}\overrightarrow{{\bf{I}}})}^{-1}\end{array}$$

where ${\mathbb{E}}({\overrightarrow{{\boldsymbol{e}}}\overrightarrow{{\boldsymbol{e}}}}^{{\rm{T}}})$ is the *N*×*N* error covariance matrix that holds the random error variance ${\sigma }_{{\varepsilon }_{i}}^{2}$ of the parent products in the diagonals and relative error covariances ${\sigma }_{{\varepsilon }_{i}{\varepsilon }_{j}}$ in the off-diagonals; $\overrightarrow{{\bf{I}}}={\left[1,\ldots ,1\right]}^{{\rm{T}}}$ is an ones-vector of length *N*; and ${\sigma }_{{\varepsilon }_{\bar{x}}}^{2}$ is the resulting random error variances of the merged estimate.

When only two groups of products are used as input (*N* = 2), it is generally assumed that the errors between them are independent. In this case, the weights are as follows:

18 $$\begin{array}{l}{\mathbb{E}}({\overrightarrow{{\boldsymbol{e}}}\overrightarrow{{\boldsymbol{e}}}}^{{\rm{T}}})=\left[\begin{array}{cc}{\sigma }_{{\varepsilon }_{1}}^{2} & 0\\ 0 & {\sigma }_{{\varepsilon }_{2}}^{2}\end{array}\right]\\ {\omega }_{1}=\frac{{\sigma }_{{\varepsilon }_{2}}^{2}}{{\sigma }_{{\varepsilon }_{1}}^{2}+{\sigma }_{{\varepsilon }_{2}}^{2}}\quad \;{\omega }_{1}=\frac{{\sigma }_{{\varepsilon }_{1}}^{2}}{{\sigma }_{{\varepsilon }_{1}}^{2}+{\sigma }_{{\varepsilon }_{2}}^{2}}\end{array}$$

In most cases, we can identify three sets of products as inputs (*N* = 3). In this scenario, we consider the possibility of error homogeneity, assuming a non-zero ECC exists between inputs 1 and 2. In this case, the error matrix can be represented as:

19 $${\mathbb{E}}({\overrightarrow{{\boldsymbol{e}}}\overrightarrow{{\boldsymbol{e}}}}^{{\rm{T}}})=\left[\begin{array}{ccc}{\sigma }_{{\varepsilon }_{1}}^{2} & {\sigma }_{{\varepsilon }_{1}{\varepsilon }_{2}} & 0\\ {\sigma }_{{\varepsilon }_{1}{\varepsilon }_{2}} & {\sigma }_{{\varepsilon }_{2}}^{2} & 0\\ 0 & 0 & {\sigma }_{{\varepsilon }_{3}}^{2}\end{array}\right]$$

The weights can then be written as:

20 $$\begin{array}{l}\overrightarrow{{\bf{W}}}=\left\{\begin{array}{c}\frac{{\sigma }_{{\varepsilon }_{2}}^{2}-{\sigma }_{{\varepsilon }_{1}{\varepsilon }_{2}}}{({\sigma }_{{\varepsilon }_{1}}^{2}{\sigma }_{{\varepsilon }_{2}}^{2}-{\sigma }_{{\varepsilon }_{1}{\varepsilon }_{2}}^{2})\ast {\mathbb{Z}}}\\ \frac{{\sigma }_{{\varepsilon }_{1}}^{2}-{\sigma }_{{\varepsilon }_{1}{\varepsilon }_{2}}}{({\sigma }_{{\varepsilon }_{1}}^{2}{\sigma }_{{\varepsilon }_{2}}^{2}-{\sigma }_{{\varepsilon }_{1}{\varepsilon }_{2}}^{2})\ast {\mathbb{Z}}}\\ \frac{1}{{\sigma }_{{\varepsilon }_{3}}^{2}\ast {\mathbb{Z}}}\end{array}\right.\\ {\mathbb{Z}}=\frac{{\sigma }_{{\varepsilon }_{1}}^{2}+{\sigma }_{{\varepsilon }_{2}}^{2}-2{\sigma }_{{\varepsilon }_{1}{\varepsilon }_{2}}}{{\sigma }_{{\varepsilon }_{1}}^{2}{\sigma }_{{\varepsilon }_{2}}^{2}-{\sigma }_{{\varepsilon }_{1}{\varepsilon }_{2}}^{2}}+\frac{1}{{\sigma }_{{\varepsilon }_{3}}^{2}}\end{array}$$

It is essential to acknowledge that before applying these weights for merging the data sets, it is necessary to address any existing systematic differences. Typically, this is achieved by rescaling the data sets to a standardized data space. Consequently, the weights can be derived from the rescaled data sets using Eqs. (2-3) and converge accordingly. This procedure ensures the accuracy and reliability of the merged data sets for further analysis.

If ECC is not considered (i.e., setting ${\sigma }_{{\varepsilon }_{1}{\varepsilon }_{2}}=0$), Eq. (20) represents the weight calculation method commonly used in most TC fusion studies. This method was initially applied by Yilmaz *et al*.^46^ in the fusion of multisource soil moisture products and later improved by Gruber *et al*.^44^ and further applied in the production of the ESA CCI global soil moisture product^47^. Dong *et al*. (2020b) also adopted this approach to fusing multisource precipitation products. In the study of evapotranspiration, Li *et al*.^22^ and Park *et al*.^15^ utilized a weight calculation method that does not consider non-zero ECC and fused multiple ET products in the Nordic and East Asia, respectively, achieving satisfactory fusion results.

In contrast to the fusion studies mentioned above, the consideration of non-zero ECC is incorporated into the fusion process and the weight calculation. Yilmaz and Crow^48^ have demonstrated that TC underestimates error variances when the zero ECC assumption is violated. Li *et al*.^36^, in their evaluation study of global transpiration products using the collocation method, also indicated the existence of error homogeneity issues between commonly used products (such as GLDAS and GLEAM), necessitating the consideration of the influence of non-zero ECC. The merging technique employed in this study provides a more explicit characterization of product errors and facilitates the derivation of more reliable weight coefficients, thereby achieving superior fusion outcomes.

The differences in results are evaluated at the site scale by contrasting the scenarios without considering non-zero ECC and directly using simple averages to compare and validate the advantages of the weight calculation method used in our study.

### Partition method

In this study, we applied three distinct methodologies to estimate transpiration (T) from eddy covariance (EC) datasets: (i) the water use efficiency (uWUE) method^49^; (ii) the Pérez-Priego method^50^; (iii) the Transpiration Estimation Algorithm (TEA) method^51^. Each of these methods provides unique insights into this crucial component of the terrestrial water cycle. We calculated the average values of these three partition methods to serve as benchmarks for validating site-scale fusion results.

These three methods exhibit disparities in their assumptions, structural design, and conceptualization disparities. These disparities encompass aspects such as the number of parameters employed (one or two in uWUE, depending on the temporal scale, versus four in Pérez-Priego), parametric versus nonparametric approaches (uWUE and Pérez-Priego versus TEA), the assumption that transpiration (T) is approximately equal to evapotranspiration (ET) for some portion of the data (uWUE and TEA versus Pérez-Priego), and the inclusion of physiological parameters characterizing leaf carbon-water optimality (Pérez-Priego and uWUE versus TEA).

Our selection of these methods is deliberate, as they are specifically designed to harness contemporary EC datasets, such as those provided by FLUXNET and its associated regional networks. These datasets are valuable due to their continuous measurements of critical variables, including CO_2_ concentrations, sensible heat flux, latent heat flux, and meteorological parameters. These variables are recorded at half-hourly or hourly intervals. Leveraging this wealth of data, all three methods rely on estimates of Gross Primary Productivity (GPP) to partition total evapotranspiration (ET) into its constituent components, namely, evaporation I and transpiration (T). This partitioning is founded on the fundamental principle that the uptake of CO_2_ and the loss of water vapor through transpiration are intricately linked processes regulated by stomatal conductance in higher plants^52^.

It is essential to acknowledge the existence of alternative approaches for ET partitioning^18,53^. While these alternative methods offer valuable tools for specialized applications, they were not subjected to detailed examination within the scope of this investigation.

### The underlying water use efficiency (uWUE) method

The uWUE method, which is the simplest of the three methods to calculate, relies on estimates of the *uWUE*, defined as,

21 $$uWUE=\frac{GPP\times \sqrt{VPD}}{ET}$$

Where VPD is the vapor pressure deficit, we have computed two variants of the uWUE metric using half-hourly data: (a) potential uWUE_p_, determined at an annual scale by establishing ^a^95th percentile regression relationship between GPP × √VPD and ET. This variant characterizes conditions where the carbon gain is maximized relative to water loss, leading to T ≈ ET; (b) apparent uWUE_a_, which is derived as the linear regression slope within a moving window spanning either one or eight days or directly from Eq. (21) when estimating at a half-hourly resolution, contingent on the desired smoothing level and data availability. In the case of uWUE_p_, it is assumed to remain constant throughout the year, aligning with the notion of maximum carbon gain to water loss, as demonstrated across a diverse range of sites and associated with stomatal optimality^54^. T/ET is then estimated as:

22 $$\frac{T}{ET}=\frac{uWU{E}_{a}}{uWU{E}_{p}}$$

### The Pérez-Priego method

The Pérez-Priego method^50^ employs a comprehens“ve “big”eaf” model incorporating four distinct parameters within a 5-day moving window. These parameters are intricately linked to the response of canopy conductance to vapor pressure deficit (VPD), photosynthetically active radiation (PAR), and temperature. Additionally, they govern the response of the maximum photosynthetic rate to VPD and ambient CO2 levels. A notable aspect of this method is its incorporation of the leaf optimality concept, wherein the maximization of carbon gain relative to water loss is a central objective. This is achieved by integrating a penalty mechanism within the cost function for parameters that yield suboptimal leaf carbon-water optimality.

The Pérez-Priego method presents a practical and physiologically grounded framework for partitioning water fluxes. It holds considerable applicability across diverse flux measurement sites, biomes, and plant functional types. Notably, this approach is a valuable complement to long-term flux measurements such as those obtained through FLUXNET. An additional advantage of this method lies in its capacity to unveil the underlying mechanisms driving plants to adapt and exhibit distinct behaviors under varying environmental conditions.

### The transpiration estimation algorithm (TEA) method

The TEA method^51^ employs a nonparametric approach, leveraging the random forest technique to forecast Water Use Efficiency (WUE), denoted as the ratio of Gross Primary Productivity (GPP) to Transpiration (T). The framework is trained on ecosystem-level WUE (*WUE*_*eco*_), the ratio of GPP to Evapotranspiration (ET), specifically during periods within the growing season and under conditions where surfaces are anticipated to dry, indicates minimal E/ET ratios. The model employs a filtering process based on precipitation input and ET values calculated within a shallow bucket water balance scheme to identify periods characterized by wet surfaces. Subsequently, the random forest model, trained on *WUE*_*eco*_ data derived from the filtered periods, is applied to predict WUE values for the entire time series under investigation:

23 $$WU{E}_{TEA}=RF\left({R}_{g},{T}_{air},RH,CSWI,GPP,\ldots \right)$$

Where *R*_*g*_ is the incoming radiation, *T*_*air*_ is the air temperature, *CSWI* is the conservative surface wetness index. *GPP* and *Tair* filters were designed to ensure plants are active while *R*_*g*_ filters remove nighttime values. The *CSWI* filter attempts to remove periods where the surface is likely wet. A critical aspect of our methodology is the selection of the 75th percentile as the optimal prediction percentile. This choice was based on rigorous evaluation against synthetic data generated by three terrestrial biosphere models, demonstrating its superior performance.

### Application of partition methods

The three methods are implemented using the Python code generously provided by Nelson *et al*.^55^, which is accessible in the associated repository located at https://github.com/jnelson18/ecosystem-transpiration, complete with a tutorial. Our approach for the uWUE method involves estimating uWUEp annually and deriving uWUEa using an 8-day moving window. In the case of the Pérez-Priego method, we perform daily parameter optimization using a 5-day moving window, which is designed to contain high-quality data. The TEA method was applied per the procedure outlined in Nelson *et al*.^51^.

It is worth highlighting that the estimation procedure employed for the Pérez-Priego method did not consistently yield satisfactory solutions for the parameters. Consequently, this occasionally results in erratic values for transpiration, thereby impeding the generation of continuous T estimates. In contrast, more extensive and robust T estimations are obtainable through the TEA and uWUE methods. As a result, we rely on the average values produced by all three methods, acknowledging that there are periods during which only the TEA and uWUE methods are applicable. This averaged dataset serves as the benchmark against which we evaluate the performance of the merged results.

### Data sources

In this study, we select three global gridded vegetation transpiration datasets from 2000 to 2020 (Table 1). Additionally, we filter a subset of sites from the global flux observation network FLUXNET and employed multiple evapotranspiration partitioning methods to calculate vegetation transpiration to comprehensively compare existing products and the performance of the fusion results. We apply Kriging spatial interpolation to downscale GLDAS-2.1 and GLEAM-3.7a from 0.25° to 0.1° and upscale PMLv2 from 0.083° to 0.1° and matched the 8-day average data to the corresponding periods, resulting in three datasets with consistent spatial and temporal resolutions for fusion.

**Table 1:** Summary of transpiration products involved.

Name	Schemes	Original Resolution (all interpolated to 0.1°)		Period
GLDAS-2.1	Noah	0.25°	3-hourly	2000-present
GLEAM-3.7a	GLEAM model	0.25°	daily	1980–2022
PMLv2-v017	Penman-Monteith-Leuning	0.083°	8-day average	2000–2020

### GLDAS

The Global Land Data Assimilation System (GLDAS) product is a land-surface simulation forced by a combination of model and observation datasets incorporating advanced and sophisticated data assimilation methodologies^56^. GLDAS runs multiple land-surface models (LSMs), including Noah, Mosaic, Variable infiltration capacity (VIC), and the Community land model (CLM). These combined models provide global evapotranspiration estimations at fine and coarse spatial (0.1° and 0.25°) and temporal (3-hourly and monthly) resolutions. The latest GLDAS version 2 has three components (v2.0-v2.2). The GLDAS-2.1 started on January 2000 to present using meteorological analysis fields from ECMWF. The transpiration parameter is derived from GLDAS-2.1 products denoted“as “TVeg_t”vg.” Transpiration is calculated as a part of total evapotranspiration using the Noah model. For more detailed descriptions of the GLDAS2 models and DA process of GLDAS-2.1, we recommend the reader refer to ‘ASA’s Hydrology Data and Information Services Center (http://disc.sci.gsfc.nasa.gov/hydrology, last access: 22 April 2024).

### GLEAM

The latest version of the Global Land Evaporation Amsterdam Model 3.7 (GLEAMv3.7) dataset^57,58^ at 0.25° is used. This version of GLEAM provides daily estimations of actual evaporation, bare soil evaporation, canopy interception, transpiration from vegetation, potential evaporation, and snow sublimation from 1980 to 2022. The third version of GLEAM contains a new DA scheme, an updated water balance module, and evaporative stress functions. Two datasets that differ only in forcing and temporal coverage are provided: GLEAMv3.7a-43-year period (1980 to 2022) based on satellite and reanalysis (ECMWF) data; GLEAMv3.7b-20-year period (2003 to 2022) based on only satellite data. The cover-dependent potential evaporation rate (*E*_*P*_) is calculated using the Priestley-Taylor equation^59^. Then, a multiplicative stress factor is used to convert *E*_*P*_ into actual transpiration or bare soil evaporation, which is the function of microwave vegetation optimal depth (VOD) and root-zone soil moisture. For detailed description, please refer to the description paper^57^. The data are freely available on the GLEAM website (https://www.gleam.eu, last access: 22 April 2024).

### PMLv2

The Penman-Monteith-Leuning version 2 global evaporation model (PMLv2) has been developed based on the Penman-Monteith-Leuning model^60,61^. The daily inputs for this model include leaf area index (LAI), white broadband albedo, and emissivity obtained from the Moderate Resolution Imaging Spectroradiometer (MODIS), as well as temperature variables (*T*_*max*_, *T*_*min*_, *T*_*avg*_), instantaneous variables (*P*_*surf*_, *P*_*a*_, *U*, *q*), and accumulated variables (*P*_*rcp*_, *R*_*ln*_, *R*_*s*_) from GLDAS2. Evaporation is divided into direct evaporation from bare soil (*E*_*s*_), evaporation from solid water sources (water bodies, snow, and ice) (*ET*_*water*_), and vegetation transpiration (*E*_*c*_). To ensure its accuracy, the PMLv2-ET model was calibrated against 8-daily eddy covariance data from 95 global flux towers representing ten different land cover types. In this study, we employ the latest version, v017. The data are freely available through Google Earth Engine (https://developers.google.com/earth-engine/datasets/ catalog/CAS_IGSNRR_PML_V2_v017, last access: 22 April 2024).

### FLUXNET

The latest FLUXNET2015 4.0 eddy-covariance data were used in our study^62^. Following the filtering process by Lin *et al*.^54^ and Li *et al*.^53^, only the measured and good-quality gap-filled data were used for quality control. Secondly, we excluded days with rainfall and the subsequent day after rainy events to mitigate the impact of canopy interception (Medlyn *et al*., 2017; Knauer *et al*., 2018). The data are freely available on the FLUXNET website (https://fluxnet.org, last access: 22 April 2024).

After data filtering and processing, 199 sites were selected. The selected sites are distributed globally, primarily in North America and Europe. The International-Geosphere–Biosphere Program (IGBP) land cover classification system^63^ was employed to distinguish the 11 Plant Functional Types (PFTs) across sites based on the classification provided in the original dataset, including evergreen needle leaf forests (ENF, 49 sites), evergreen broadleaf forests (EBF, 13 sites), deciduous broadleaf forests (DBF, 26 sites), croplands (CRO, 20 sites), grasslands (GRA, 39 sites), savannas (SAV, 8 sites), mixed forests (MF, 8 sites), closed shrublands (CSH, 2 sites), open shrublands (OSH, 13 sites), and permanent wetland (WET, 16 sites).

## Data Records

The dataset is available at figshare^64^. The daily data for each year are stored in individual netCDF files following the naming convention “Merged.Tveg.yyyy.nc”, where, for example, the data for the year 2002 are stored in the file “Merged.Tveg.2002.nc”. Each netCDF file contains four variables: “lat” (standard name: latitude), “lon” (standard name: longitude), “day” (standard name: day of the year) and “Ec” (standard name: vegetation transpiration). We have also provided MATLAB code (“readdata_example.m”) for data retrieval and visualization as a reference for users.

## Technical Validation

In this study, we aim to meticulously assess the performance of fused products at both site-specific and global scales. We evaluate the fused products at the site level by comparing them against mean transpiration (T) estimates obtained through three partitioning methods at selected FLUXNET sites. These assessments are further juxtaposed with other product variants, including simple averages and conditions that omitted considering non-zero eddy covariance correction (ECC). We scrutinize the spatial variations in land surface transpiration computed by the fused products globally, drawing comparisons with input results.

### Analysis of error variances and weights

We first analyse the random error variance of the input products computed using the EIVD method. Here, we assumed a scenario where random errors were homoscedastic between GLDAS and GLEAM. The remaining potential ECC scenarios were also calculated using the EIVD method and are analysed in the discussion section.

**Fig. 1:**
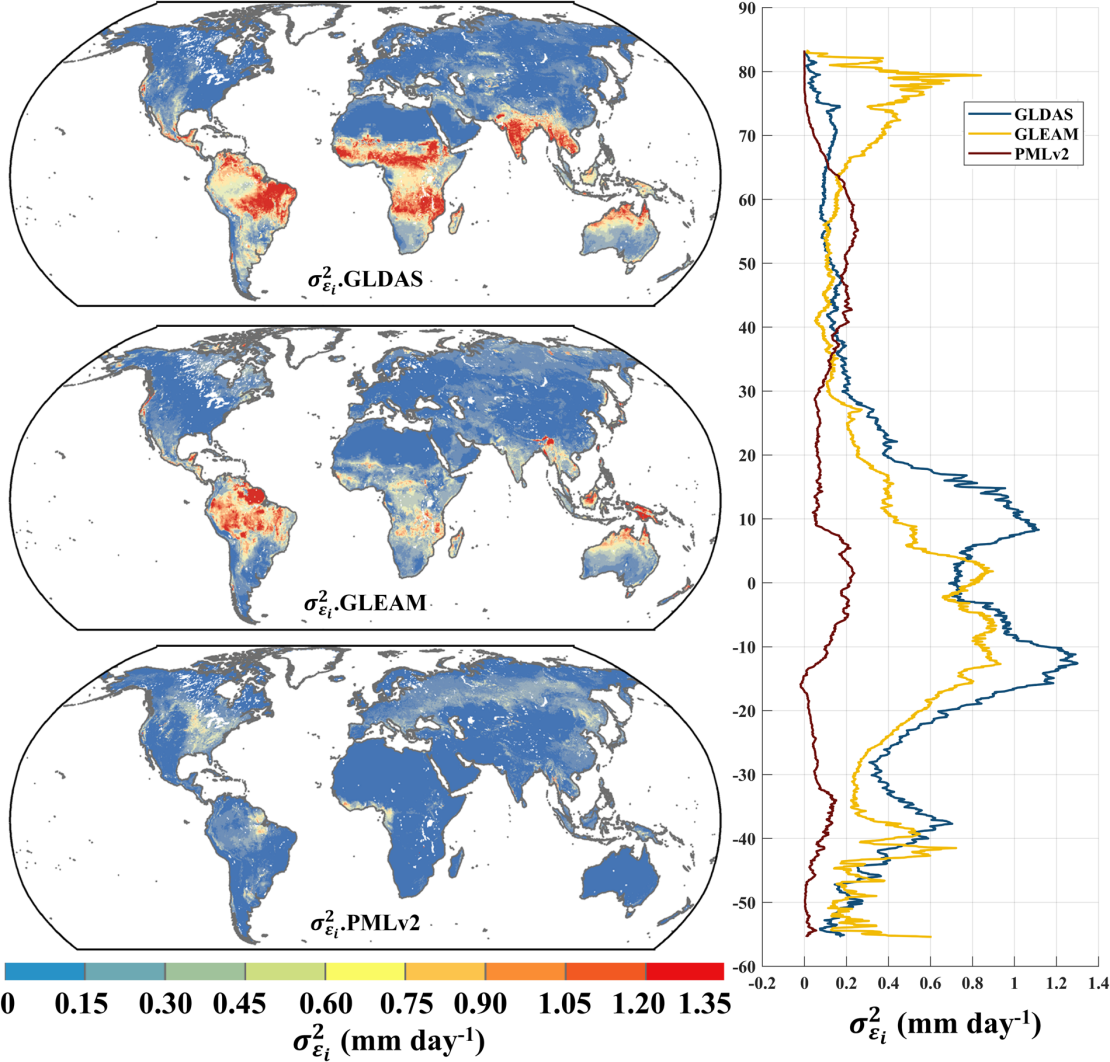
Global distribution of random error variances (${\sigma }_{{\varepsilon }_{i}}^{2}$) of GLDAS, GLEAM, and PMLv2 using EIVD at 0.1° from 2000 to 2000, depicted alongside corresponding variation curves of the average ${\sigma }_{{\varepsilon }_{i}}^{2}$ with latitude.

Figure 1 depicts the random errors of the products calculated using the EIVD method from 2000 to 2020 at 0.1°, where a non-zero ECC is assumed between GLDAS and GLEAM. The global random error variances (mean ± standard deviation) obtained using the EIVD method were as follows: GLDAS: 0.36 ± 0.43 mm day^−1^, GLEAM: 0.29 ± 0.35 mm day^−1^, PMLv2: 0.13 ± 0.16 mm day^−**1**^. These results indicate that PMLv2 performed best overall, while GLDAS performed the poorest. Regarding the global spatial distribution, GLDAS exhibit high random errors in Central South America, Southern Africa, Southeast Asia, and South Asia. GLEAM similarly showed poorer performance in Central South America and Indonesia.

In contrast, PMLv2 demonstrates minor errors on a global scale, particularly excelling in the Amazon region. Li *et al*.^36^ analyse the performance of GLDAS and GLEAM at a 0.25° resolution using the EIVD method. Although they employ different triplets to calculate the EIVD results, the spatial distribution of random errors for GLDAS and GLEAM obtained in their study are similar, indicating that GLDAS exhibits more significant errors than others.

The latitudinal distribution reveal that overall, PMLv2 outperforms GLEAM and GLDAS. There could be two reasons for this phenomenon: (1) PMLv2 employs a transpiration calculation model that considered vegetation stomatal conductance, offering a more physically grounded approach. Additionally, it utilizes observational data from flux stations for calibration and correction, providing a more robust physical basis^60^; (2) In this study, the data from GLDAS and GLEAM are interpolated from 0.25° to 0.1°, which involves a straightforward statistical downscaling process that may introduce some uncertainty. This aspect will be discussed further in the subsequent sections.

**Fig. 2:**
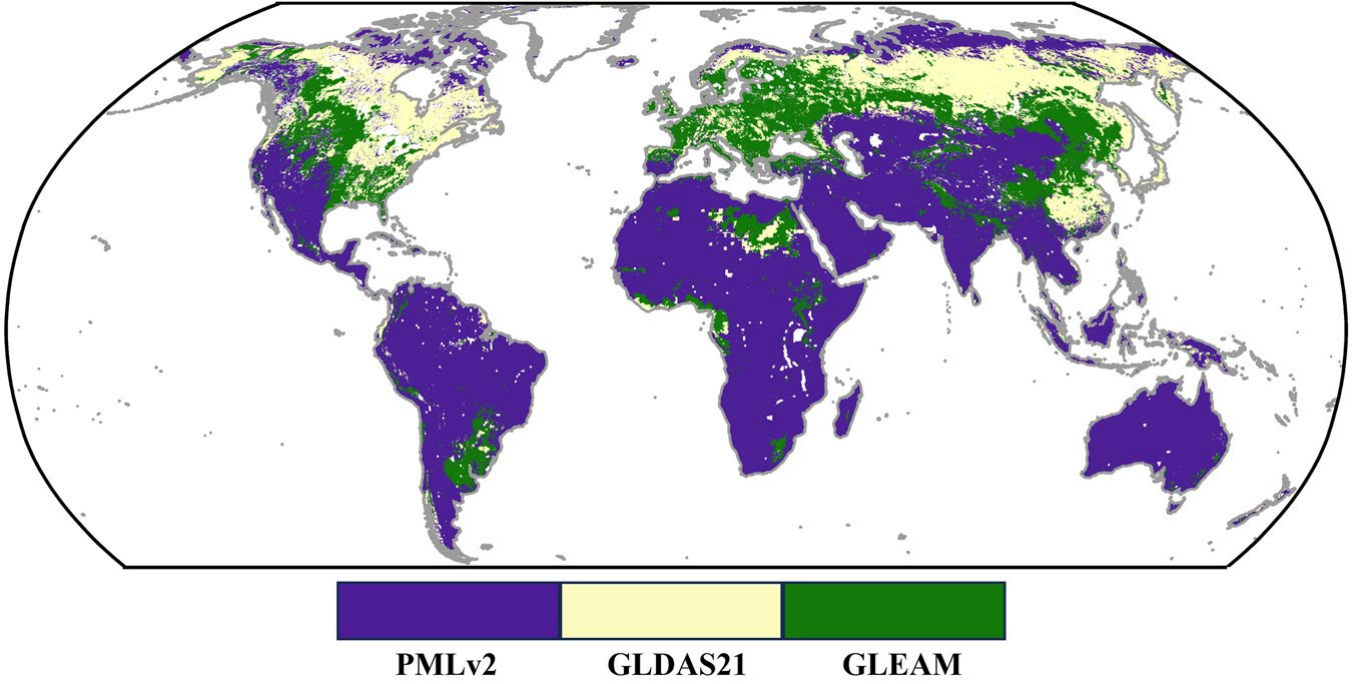
Map of the prevailing product at individual pixels based on weights.

Next, in Fig. 2, we present the dominant product for each grid cell, where dominance refers to the product with the highest assigned weight. The results in Fig. 2 indicate that the weights for PMLv2 and GLEAM are higher than ERA5L, aligning with the error calculations presented in Fig. 1. PMLv2 predominantly covers the Southern Hemisphere, while GLEAM and GLDAS dominate different regions over the Northern Hemisphere. This underscores the effectiveness of error and weight analysis based on collocation in reflecting product performance, thereby allowing for a rational adaptation of weights. The error and weight computation methods based on collocation can only provide the minimum MSE solution for a given combination of inputs. It is important to note that changes in inputs will impact the results.

### Site-scale evaluation

**Fig. 3:**
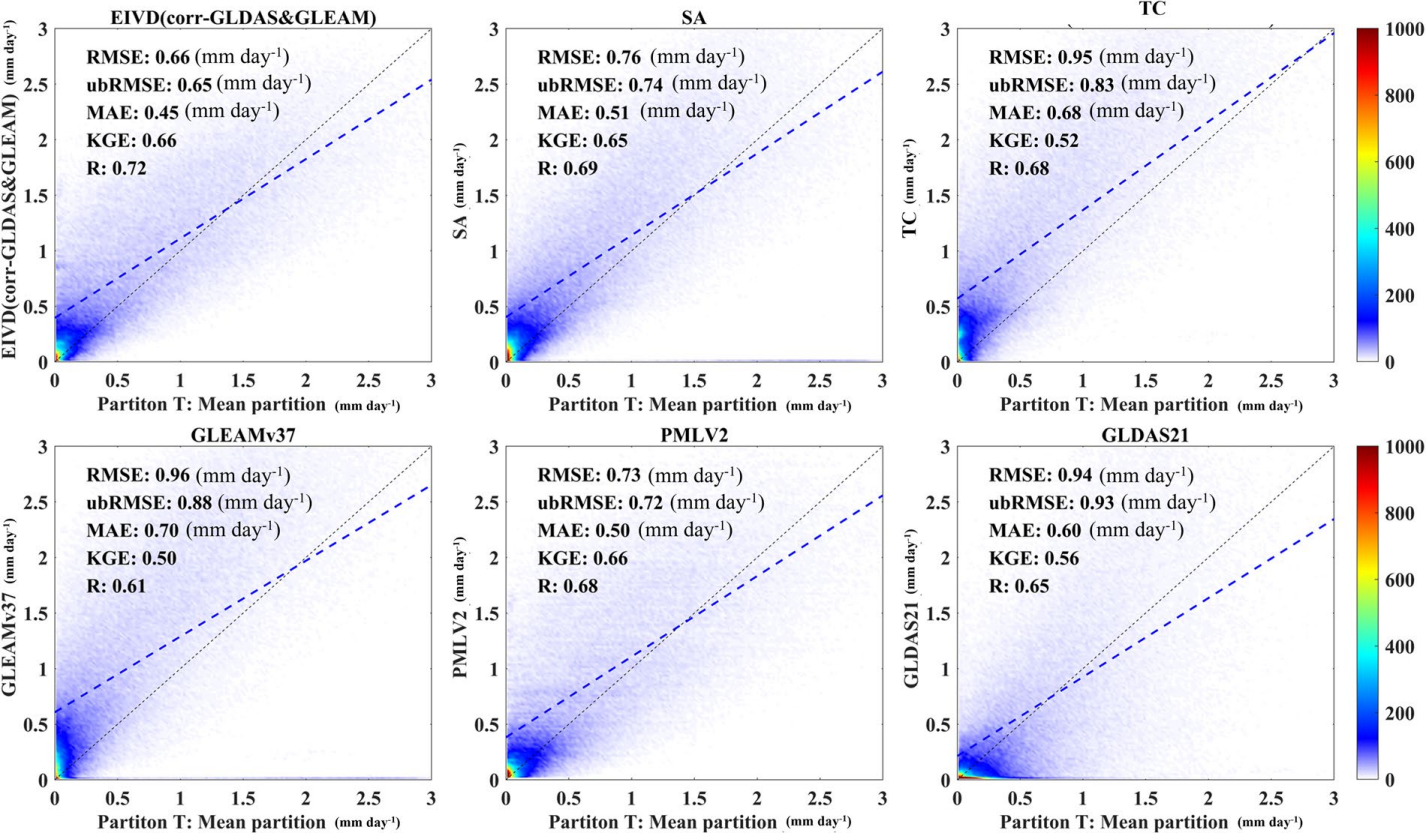
Scatter plots of products corresponding to the available period data from FLUXNET sites. The color bar represents the density, with darker color indicating higher concentration, with “SA” indicating the results based on the simple average.

**Fig. 4:**
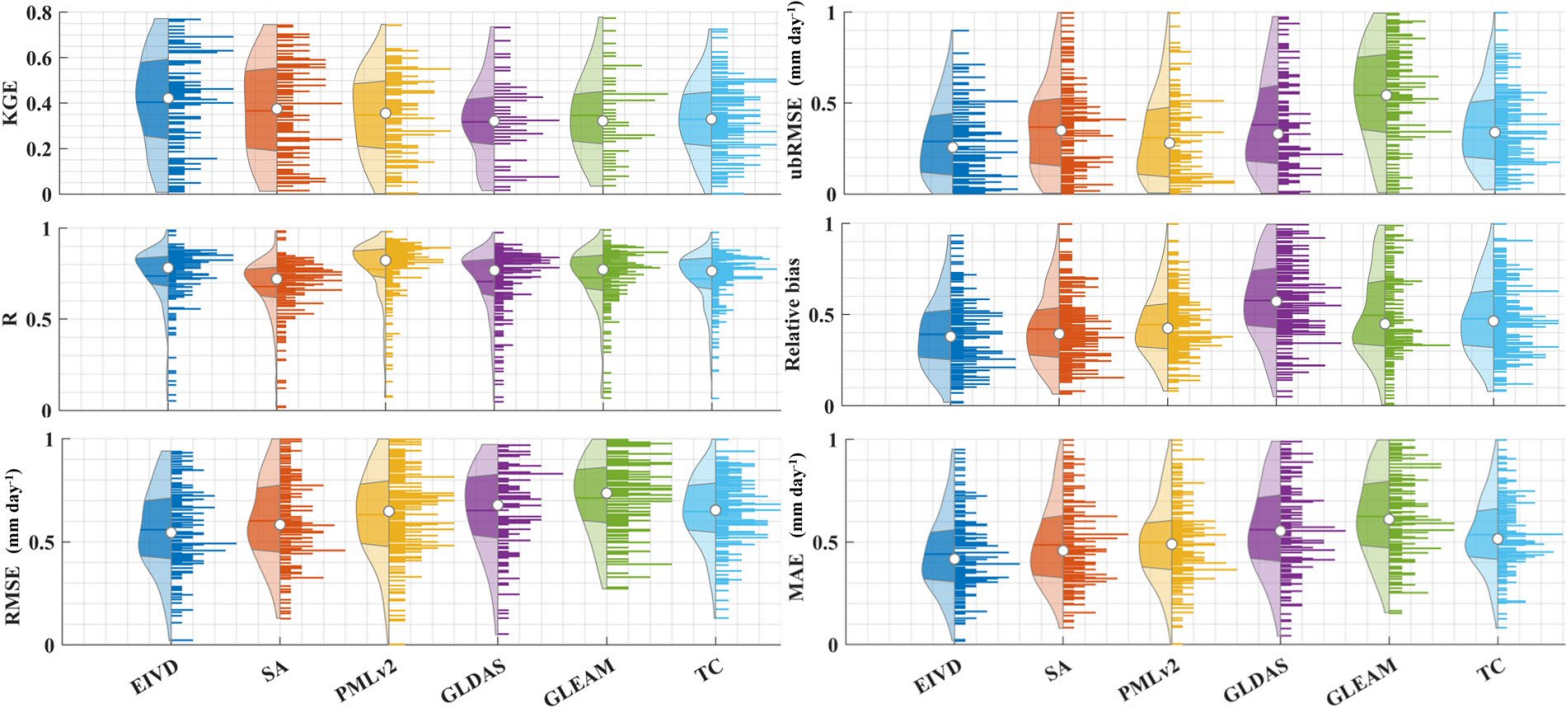
Violin plots obtained by aggregating five different statistical indicators, calculated separately for each site. In each violin plot, the left side represents the distribution, with the shaded area indicating the box plot, the dot representing the mean, and the right side showing the histogram.

At the site scale, this study uses the average transpiration values calculated using three different ET partitioning methods as benchmarks to evaluate the performance of the fused products. Simultaneously, the results are compared between three input data sets and TC-merged results (without considering non-zero ECC conditions). Figure 3 corresponds to Table 2, where statistical parameters are computed by pooling data from all sites. Similarly, Fig. 4 corresponds to Table 3, where statistical parameters are computed separately for each site and then analysed.

**Table 2:** Average values (mean ± standard deviation) of different metrics of all sites.

Product	RMSE (mm day^−1^)	ubRMSE (mm day^−1^)	MAE (mm day^−1^)	RB	KGE	R
EIVD-Merged	0.66 ± 0.17	0.65 ± 0.16	0.45 ± 0.22	0.32 ± 0.16	0.66 ± 0.14	0.72 ± 0.13
TC-Merged	0.95 ± 0.23	0.83 ± 0.16	0.68 ± 0.34	0.37 ± 0.21	0.52 ± 0.15	0.68 ± 0.18
SA	0.76 ± 0.24	0.74 ± 0.25	0.51 ± 0.25	0.41 ± 0.12	0.65 ± 0.16	0.69 ± 0.19
GLDAS	0.94 ± 0.22	0.93 ± 0.13	0.60 ± 0.18	0.35 ± 0.20	0.56 ± 0.08	0.65 ± 0.11
GLEAM	0.96 ± 0.19	0.88 ± 0.21	0.70 ± 0.24	0.34 ± 0.15	0.50 ± 0.11	0.61 ± 0.15
PMLv2	0.73 ± 0.14	0.72 ± 0.15	0.50 ± 0.24	0.35 ± 0.23	0.66 ± 0.13	0.68 ± 0.17

**Table 3:** Average values (mean ± standard deviation) of indicators corresponding to different products, calculated based on the comprehensive results obtained for each site.

Product	RMSE (mm day^−1^)	ubRMSE (mm day^−1^)	MAE (mm day^−1^)	RB	KGE	R
EIVD-Merged	0.56 ± 0.18	0.29 ± 0.16	0.44 ± 0.21	0.39 ± 0.11	0.40 ± 0.15	0.74 ± 0.24
TC-Merged	0.65 ± 0.21	0.37 ± 0.22	0.56 ± 0.33	0.48 ± 0.14	0.33 ± 0.14	0.72 ± 0.15
SA	0.60 ± 0.28	0.38 ± 0.14	0.49 ± 0.25	0.42 ± 0.30	0.37 ± 0.11	0.68 ± 0.17
GLDAS	0.71 ± 0.25	0.31 ± 0.15	0.62 ± 0.41	0.58 ± 0.28	0.32 ± 0.11	0.71 ± 0.12
GLEAM	0.65 ± 0.18	0.37 ± 0.16	0.54 ± 0.30	0.49 ± 0.18	0.36 ± 0.14	0.73 ± 0.14
PMLv2	0.63 ± 0.17	0.54 ± 0.27	0.50 ± 0.27	0.44 ± 0.25	0.35 ± 0.13	0.77 ± 0.13

The findings depict in Fig. 3 underscored the enhanced accuracy achieved by the EIVD method in transpiration estimation through fusion. Notably, the fusion outcomes exhibited marked improvements across multiple parameters compared to three sets of products. These improvements are evident in correlation metrics, where the Pearson coefficient for the fusion results reached 0.72 ± 0.13, and the KGE registered at 0.66 ± 0.14, surpassing the input data’s performance. Error metrics also reflect these advancements, with the fusion results displaying lower RMSE, ubRMSE, MAE and RB values than the input datasets.

However, it can also be seen that EIVD-based results at some sites exhibit RMSE over 0.70 mm day^−1^ (with a maximum of 0.83 mm day^−1^), along with the relative errors of approximately 40–60% (with a maximum of 48%). This discrepancy may arise from systematic errors inherent in the fusion method and errors associated with the three ET partitioning methods used as benchmarks. Further research is warranted to enhance transpiration estimates, address methodological variations, and reduce uncertainties associated with ET partitioning methods. Nonetheless, despite these challenges, applying the EIVD method in fusion mitigated errors associated with the inputs, yielding more promising outcomes when compared with individual inputs.

These findings indicate that applying the EIVD method in fusion effectively mitigated errors associated with the inputs, resulting in more promising outcomes.

Furthermore, when comparing the results of the EIVD method with those of the Simple Average (SA) and TC fusion methods, all three fusion approaches exhibit enhancements in correlation metrics (as indicated by KGE and R). However, the EIVD method notably reduce errors, including RMSE and other measures. EIVD fusion results are superior to TC fusion results, suggesting that considering non-zero ECC was meaningful for fusion based on collocation analysis. Additionally, while the SA method could achieve decent fusion results, it is observed that a significant portion of points clustered around the x-axis. This clustering phenomenon may be attributed to the prevalence of estimation values close to zero in GLDAS or GLEAM. Consequently, these findings indicate that the SA method did not emerge as the optimal fusion approach in this study, with the EIVD method proving to be a more reliable alternative.

The information in Table 2 corresponded to Fig. 3, with the bolded sections corresponding to the products that performed the best in their respective statistical metrics. The results indicate that the fused data obtained in this study showed strong performance across various indicators at the site scale when averaging using three ET partition methods as a benchmark. Furthermore, the performance of PMLv2 is notably impressive. Although this product solely used FLUXNET data for ET correction^60^, it theoretically enhances its transpiration estimation, which could potentially explain the strong performance observed in the PMLv2 product.

Figure 4 corresponds to Table 3. Statistical parameters are computed separately for each site, and the results are used to generate violin graphs. Our findings demonstrate that, within the scope of this study, the fused transpiration product consistently outperforms the three sets of inputs, as well as the simple average (SA) and TC methods, across a diverse range of performance metrics. Regarding correlation metrics, the fused product exhibits higher KGE scores than the other products and combinations. While the R index is slightly lower when compared to the PMLv2 method, this aligned with the observed trends in Fig. 3. When examining error metrics, the fused results consistently exhibit promising performance. However, the evaluation results of relative bias also indicate that there is still some bias (0.39 ± 0.11) compared to the results from site-level ET partitioning. This discrepancy may be attributed to systematic errors in the fusion method or errors inherent in the ET partitioning methods. Further efforts are required to enhance transpiration estimates, address the variability among different methods, and reduce uncertainties associated with ET partitioning methods.

Table 3 presents the average results of different statistical indicators, with the corresponding optimal products highlighted in bold. The fusion results demonstrated promising performance across all indicators, especially in reducing overall errors, as indicated by the error metrics.

**Fig. 5:**
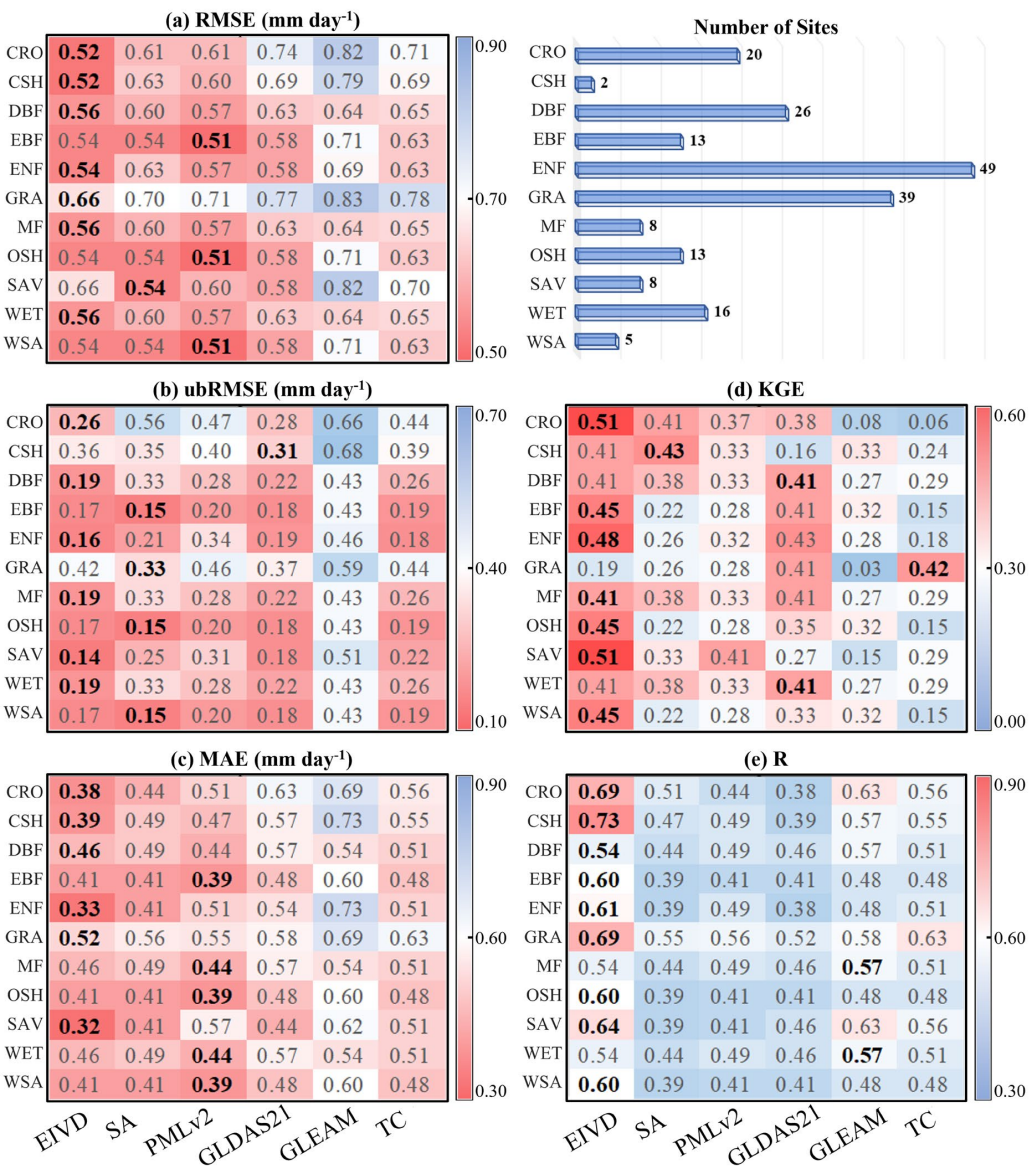
Average value of five statistical indicators for FLUXNET sites classified by PFTs. Each row represents the value for the merged product, simple-averaged (SA) result, three input datasets and TC-based results at relative sites with the same PFT. The bold value indicates the best performance for the relative indicator.

Additionally, we calculate statistical parameter averages for sites with the same PFTs based on information from 199 FLUXNET site data sources and generated a heatmap, as shown in Fig. 5. The results show that the merged product performed the best in almost all PFT categories, as indicated by various indicators. While on sites where other products perform better, merged-product indicators are comparable to the optimal products, albeit slightly inferior. This indicates that our fusion approach effectively combines the advantages of different products, resulting in superior fusion results across different vegetation types.

**Fig. 6:**
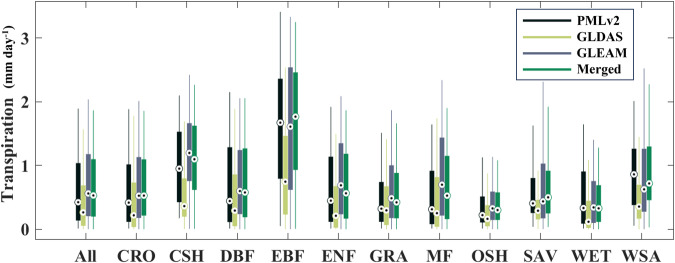
Box plots of daily transpiration estimates of three input datasets and the merged product at the same PFT sites from 2000 to 2020.

The estimation results of different products at the same PFT site are further analysed and shown in Fig. 6. It can be observed that GLDAS consistently shows lower values compared to PMLv2 and GLEAM at all sites, especially at EBF and CSH sites, with the median values differing by more than twice. The fused results obtain in this study generally fall between PMLv2 and GLEAM at most sites, with relatively higher values at EBF sites. There are significant differences among the different products.

In summary, our study, founded on the computation of average transpiration values through three distinct ET partitioning methods across 199 FLUXNET sites, entailed a comprehensive benchmark analysis of the merged outcomes. This analysis convincingly established the robust alignment of our merged results with the reference data. Furthermore, through meticulous product performance comparisons at each site, we underscore the accuracy and minimal errors associated with our merged results. These findings highlight that our merged outcome consistently exhibits equivalent or slightly enhanced precision compared to existing products, including those grounded in simple averaging techniques and TC-merged approaches.

### Global comparison

This section compares the global variation of transpiration between the merged results and the inputs. The presented results are derived from calculations utilizing data from 2000 to 2020 to ensure consistency.

**Fig. 7:**
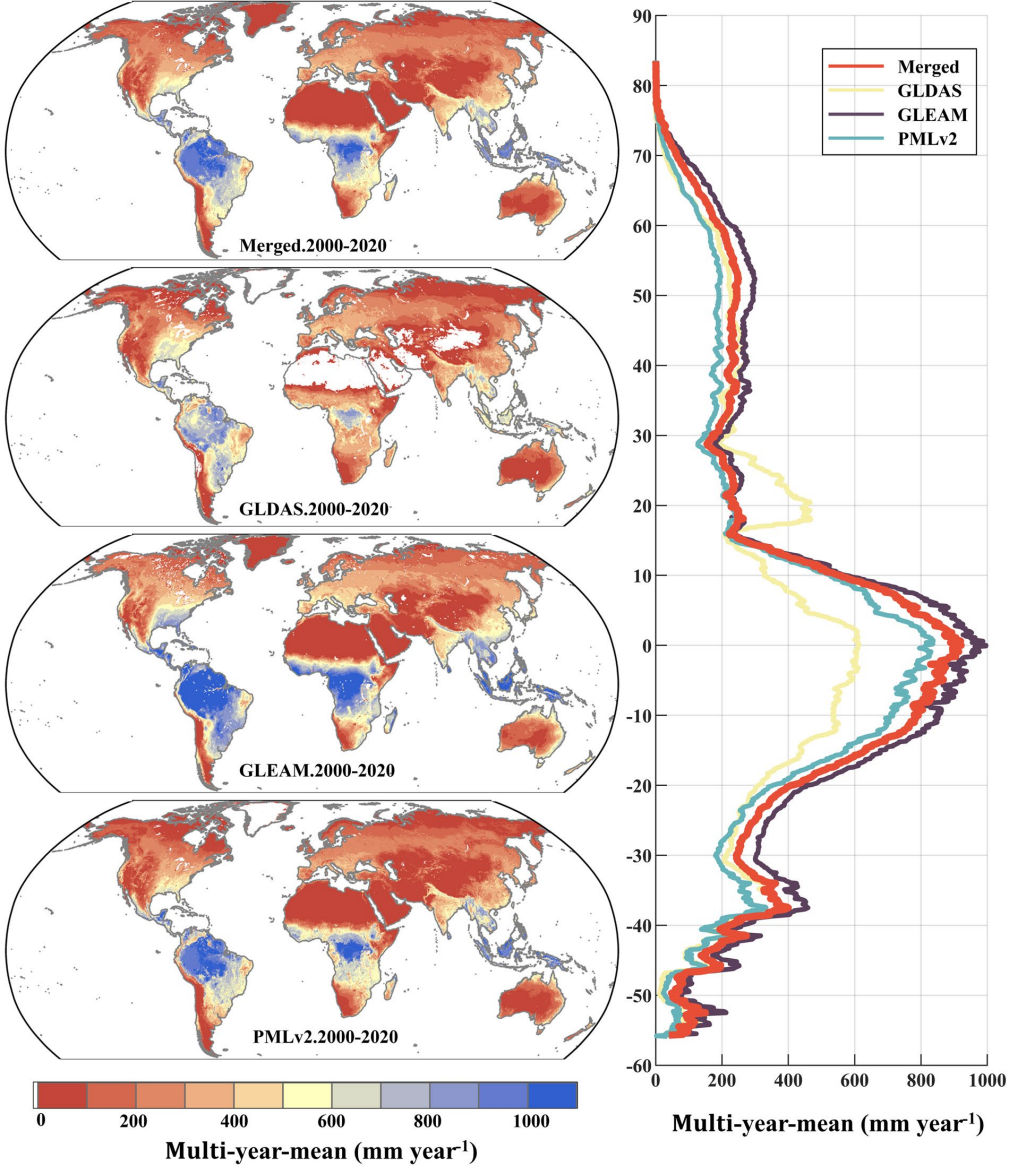
Global distribution of multiyear annual average transpiration at 0.1° for EIVD-merged results, GLDAS, GLEAM and PMLv2, depicted alongside corresponding variation curves of the average annual value with latitude.

The results in Fig. 7 indicates significant differences in the multiyear daily average distribution of global transpiration among different products, reaffirming the imperative need for data fusion. The multiyear annual transpiration results for different products are as follows (mean ± standard deviation): Merged result: 276.46 ± 106.63 mm year^−1^, GLEAM: 278.49 ± 123.31 mm year^−1^, GLDAS: 213.98 ± 73.96 mm year^−1^, and PMLv2: 207.54 ± 103.03 mm year^−1^.

Across varying products, long-term average transpiration exhibits relatively consistent variations with latitude, with the merged dataset generally tracking with PMLv2 and GLEAM. However, GLDAS exhibits notably diminished values in equatorial regions compared to other products, including the fused results. Spatial analysis further unveils that GLDAS consistently provided the lowest estimates for transpiration in tropical regions among the various product sets. Conversely, GLDAS values appear elevated near 20°N, a phenomenon potentially attributed to the absence of estimates for the Sahara Desert and the Arabian Peninsula. Consequently, this data gap increased means along this latitude relative to other products. A parallel overestimation by GLDAS is discernible near the 60°N latitude, with concurrent data gaps evident in Central Asia at this latitude.

In terms of estimating multiyear trends and seasonality, we first validate and compare the performance of the merged results with other products at the site scale. Due to the inconsistent long-term observation of FluxNet sites, trends at many sites are not significant. Therefore, we deliberately select 13 sites with continuous observations for the same 11-year period (2004 to 2014) and with significant trends. The annual transpiration values for each year are calculated as the mean of the 13 sites for that year, allowing the computation of linear trends and seasonality. We employ singular spectrum analysis (SSA), which assumes an additive decomposition A = LT + ST + R. In this decomposition, LT represents the long-term trend in the data, ST is the seasonal or oscillatory trend, and R is the remainder.

**Fig. 8:**
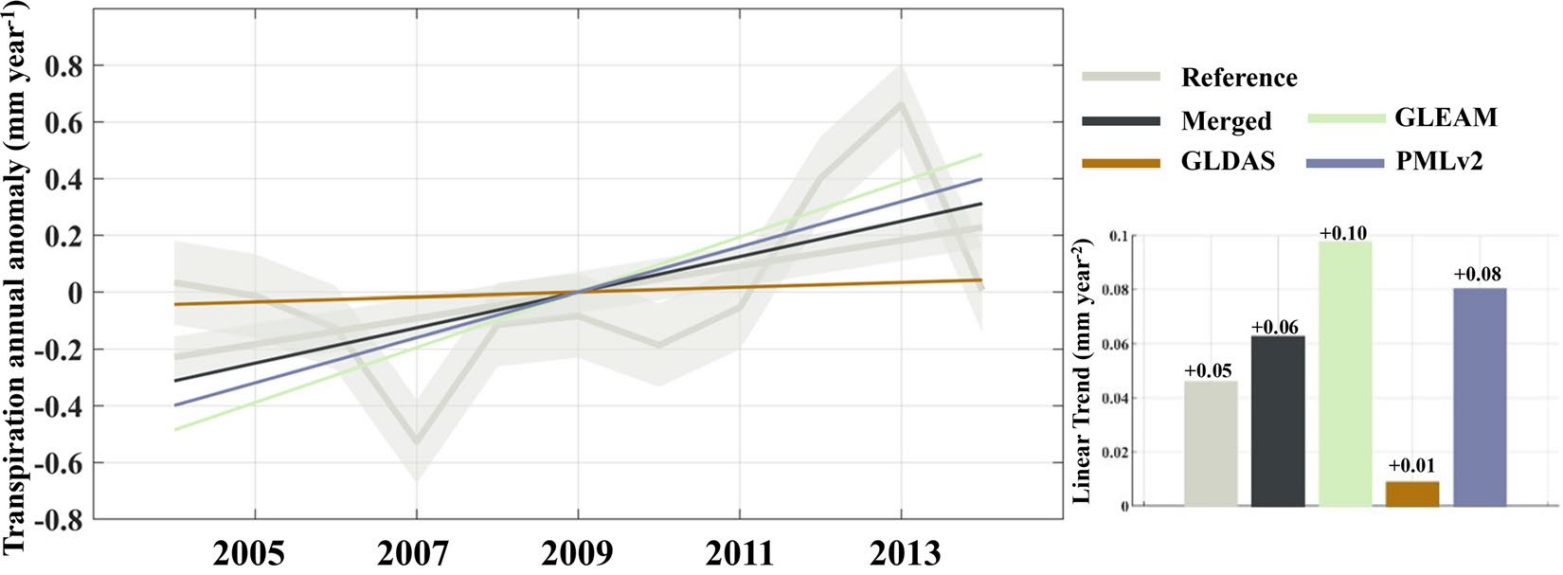
Comparison of the linear trend from 2004 to 2014 among 13 FluxNet sites using between the Merged and the inputs. The trends have been subjected to SSA decomposition, removing seasonality. The grey enveloping line represents the mean plus the standard deviation of the 13 sites.

**Fig. 9:**
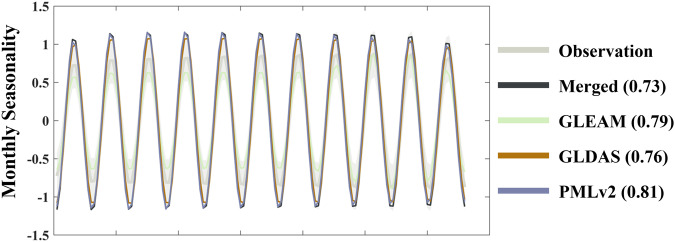
Comparison of monthly seasonality from 2004 to 2014 among 13 FluxNet sites using between the Merged and the inputs. The seasonality has been subjected to SSA decomposition, with the grey area representing the observed values. The parentheses in each product name indicate the KGE coefficient compared with the observed values.

In Figs. 8, 9, based on observations from FluxNet sites, we analyse the performance of the merged results and other products in estimating the linear trend and seasonality of transpiration over multiple years. It is important to note that we only present the analysis results for 13 sites with continuous 11-year observations, and the performance of different transpiration products in trend estimation at individual sites still varies, not fully reflecting the overall performance on all grids in terms of trend and seasonality. Nevertheless, such a comparison can still provide valuable insights.

Examining the results of the linear trend, both GLEAM and PMLv2 exhibit a significant upward trend, above the observations. While GLDAS show a milder trend, while the merged results demonstrate a gradual upward trend closer to the observations. For the analysis of seasonality, the KGE index comparing each product’s results with observed values is provided in parentheses next to the product name. Generally, all the products exhibit a good representation of seasonal variations.

Furthermore, we present site-wise comparison in Table 4. The results indicate that, the trends estimated by the merged results are consistent with the observed trends, with minor differences. In comparison to the observed monthly seasonality, although the merged results do not always prevail, the KGE values exceed 0.5 at all the sites, with some sites exceeding 0.7, indicating that it can effectively capture the seasonal variations.

**Table 4:** Comparison of the merged results and the inputs at 13 continuous 10-year observational sites.

Site_name	Linear Trend (mm year^−2^)					KGE of monthly seasonality			
	Observed	Merged	GLEAM	GLDAS	PMLv2	Merged	GLEAM	GLDAS	PMLv2
BE_Lon	0.07	0.05	0.03	0.06	0.05	0.86	0.63	0.71	0.73
CH_Lae	0.11	0.09	0.02	−0.03	−0.06	0.80	0.75	0.45	0.44
CH_Oe2	0.05	0.02	−0.02	−0.04	0.05	0.67	0.56	0.58	0.68
CZ_BK1	−0.04	−0.08	−0.03	−0.02	−0.07	0.85	0.57	0.43	0.58
DE_Gri	0.11	0.10	0.05	−0.03	−0.01	0.72	0.60	0.53	0.56
DE_Kli	−0.12	−0.15	−0.01	0.02	−0.06	0.85	0.66	0.60	0.56
FR_Gri	−0.08	−0.11	0.01	0.05	0.00	0.78	0.70	0.52	0.89
GF_Guy	0.10	0.07	0.00	−0.04	0.04	0.72	0.80	0.78	0.60
IT_BCi	0.15	0.13	0.03	0.04	0.02	0.79	0.82	0.66	0.44
IT_Noe	0.10	0.07	0.00	0.02	0.03	0.92	0.89	0.46	0.82
US_GLE	0.16	0.12	−0.03	−0.01	0.05	0.64	0.90	0.88	0.54
US_SRM	0.08	0.04	−0.03	−0.03	0.03	0.79	0.87	0.86	0.37
US_Wkg	0.17	0.13	−0.02	0.00	−0.01	0.69	0.24	0.66	0.75

(a) Comparison of linear trends. (b) KGE values for monthly seasonality.

**Fig. 10:**
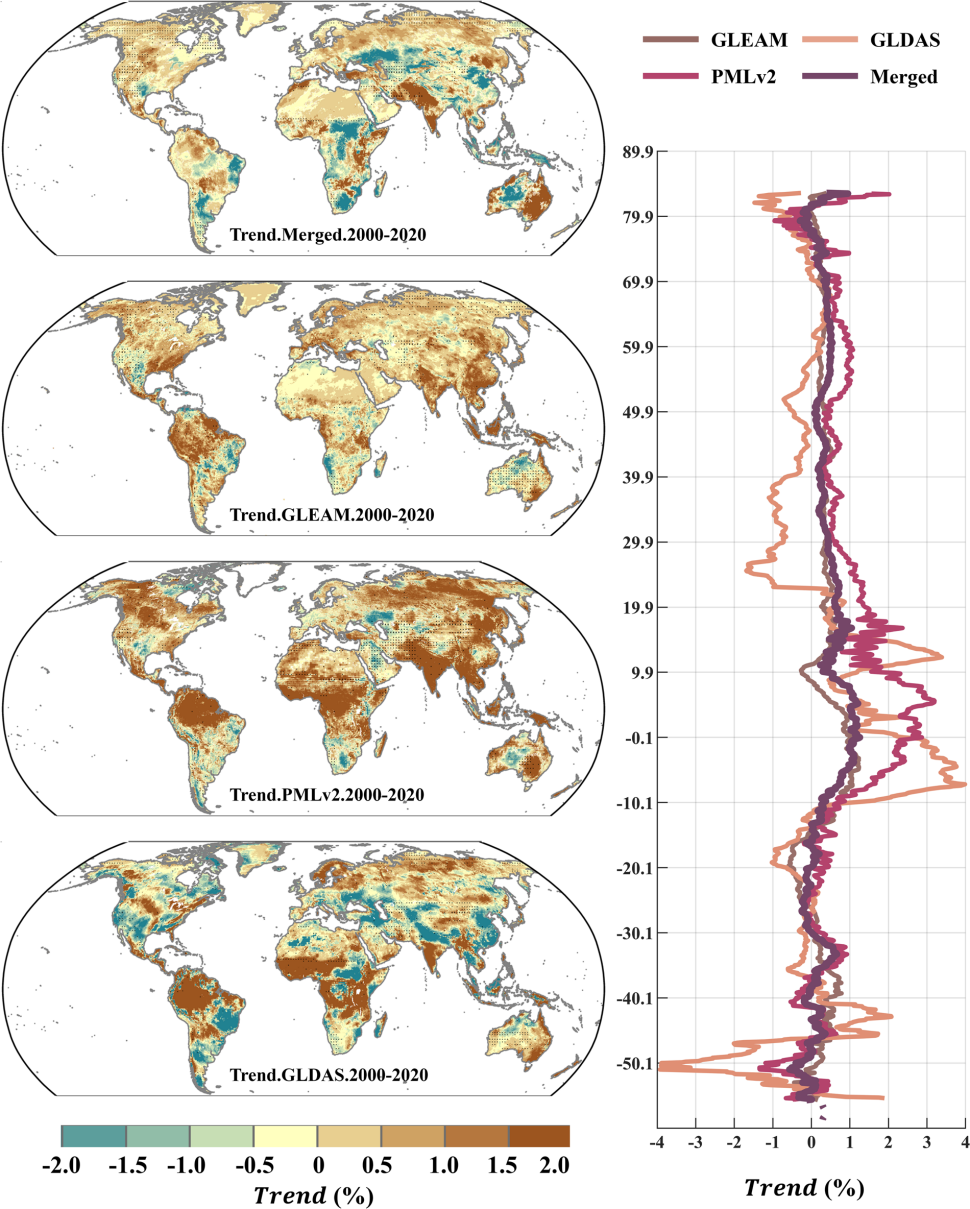
Global distribution of the multiyear linear trend for EIVD-merged results, GLEAM, GLDAS, and PMLv2, depicted alongside the corresponding average trend with latitude. The trend is estimated with the Theil–Sen slope method, and the significance level is tested with the Mann–Kendall method. The dotted area indicates that the trend has passed the significance test at the 5% level.

We further generated global maps of multiyear linear trends in transpiration, estimating trends using the Theil–Sen slope method and testing significance with the Mann–Kendall method. The dotted areas indicate trends passing a significance test at a 5% level. Figure 10 presents the linear trends of multiyear annual-scale transpiration calculated for different products at 0.1 from 2000 to 2020. The corresponding latitude-dependent variations of the rate of change are shown on the right side. It can be observed that the differences in linear trends among the different products are more significant than the multiyear averages, and in some regions, they even exhibit opposite trends.

In the Amazon rainforest region, the results of all products consistently indicate an increase in transpiration. However, the growth rate reflected by PMLv2 and GLDAS is notably higher than that of the merged product and GLEAM. In the Congo Basin, while the merged product and GLDAS show a slight decrease in transpiration, the trend indicated by PMLv2, and GLEAM is the opposite. In the tropical rainforest region of Indonesia, except for GLDAS, all other products show an increase in vegetation transpiration.

Table 5 presents the multiyear average transpiration calculated by different products and their change trends. Although the multiyear average transpiration calculated by different products is relatively close, there are significant differences in the multiyear trends. Among them, the linear trend reflected by GLDAS is significantly higher than that of other products, while the merged product is closer to PMLv2 and GLEAM. This further emphasizes the importance of effectively reducing product errors and conducting model improvements and data fusion.

**Table 5:** Comparison of average multiyear annual transpiration (mean ± standard deviation) with linear trend between the merged results and the inputs at global scales from 2000 to 2020.

	Mean annual transpiration (mm year^−1^)	Linear trend (%)
EIVD-Merged	276.46 ± 106.63	0.32
GLEAM	278.49 ± 123.31	0.27
GLDAS	213.98 ± 73.96	0.75
PMLv2	207.54 ± 103.03	0.34

In summary, the multiyear average annual transpiration data derived from the merged results demonstrated a reasonable alignment with spatial distribution patterns and latitude-related trends. In addition, site-scale assessment indicates that the merged product can effectively capture the multiyear linear trend and seasonality of transpiration, highlighting the robustness of the fusion methodology in addressing discrepancies among different product sources.

### Improved data fusion with ECC consideration

This study employs a collocation analysis-based multisource merging approach that considers non-zero ECC, a critical factor often overlooked in previous fusion methods^15,22^. Our comparative analyses, encompassing both theoretical and empirical assessments, unequivocally demonstrate the profound impact of ECC inclusion on enhancing the reliability of merged data compared to the conventional TC method. Theoretical underpinnings of collocation analysis, which evaluate the similarity among triple or quadruple inputs through cross-correlation metrics, play an important role in quantifying product errors, particularly in the absence of ground truth. The presence of familiar sources of random errors among inputs can lead to undesirable interference^48^, particularly in the context of multisource data weight calculations, resulting in heightened uncertainties.

Non-zero ECC conditions introduce more substantial bias in the results mainly due to two reasons: (1) they cannot be mitigated by rescaling; (2) they cannot be compensated even with equal magnitude for all inputs; and (3) they have been frequently reported in recent studies for various variables^21,28,41^. Within our site-scale analysis, we compared merging techniques employing EIVD and TC methodologies (Fig. 3 and Fig. 4). The improvement in merging outcomes and the substantial reduction in product errors underscore the profound significance of considering ECC. It is worth noting that while this study assumed the existence of non-zero ECC conditions between GLEAM and GLDAS, it is plausible that non-zero ECC conditions also exist between other pairs. Consequently, we present the EIVD-based ECC results for various pairs, highlighting our findings’ broader applicability and impact.

**Fig. 11:**
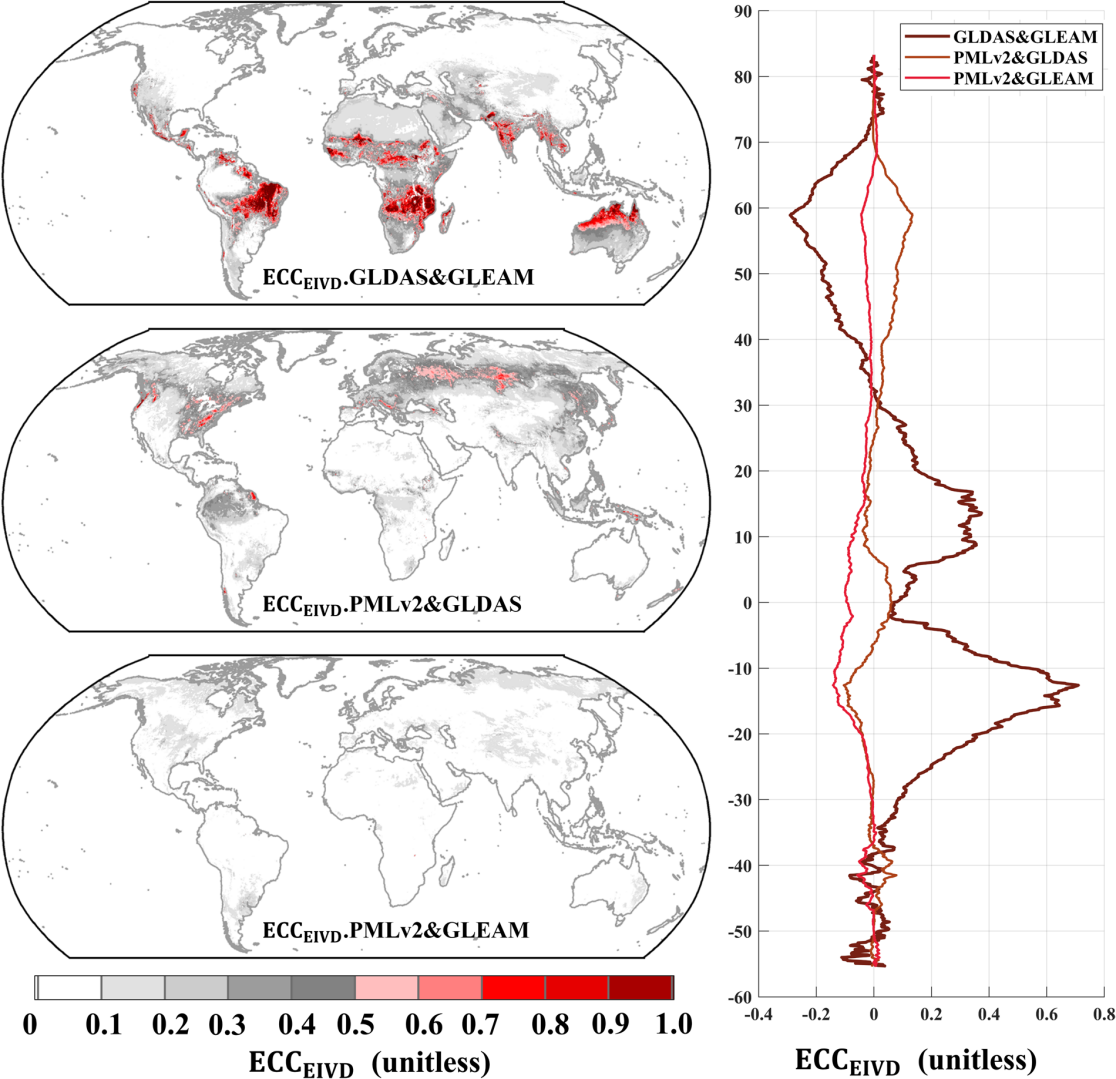
Global distribution of estimated error cross-correlation (ECC) between GLDAS, GLEAM, and PMLv2 pairwise using EIVD alongside relevant variation curves of the average ECC with latitude.

As depicted in Fig. 11, the ECC values of GLDAS and GLEAM are notably higher than those of PMLv2-GLDAS and PMLv2-GLEAM. The global average ECC values for different pairs are as follows (mean ± standard deviation): GLDAS-GLEAM: 0.22 ± 0.30, PMLv2-GLEAM: 0.06 ± 0.10, and PMLv2-GLDAS: 0.08 ± 0.13. The results of the ECC indicate the presence of correlated random errors between GLEAM and GLDAS. These errors arose from the shared utilization of driving data, such as radiation and air temperature data sourced from ERA-Interim and ESA CCI SM v2.3 soil moisture data, contributing to the observed correlation between these two products. In contrast, ECC values for PMLv2 concerning GLDAS and GLEAM are relatively small. Some error correlation is noted between PMLv2 and GLDAS, likely due to using GLDAS2.0 data in the PMLv2 driver, while this study employs GLDAS2.1 data. Importantly, considering that the mean ECC between PMLv2 and GLDAS was less than 0.1, it can be reasonably inferred that this correlation had an insignificant impact on the results of the collocation analysis.

To summarize, the results of the ECC analysis support the assumption of non-zero ECC between GLDAS and GLEAM in this study. This finding underlines the robustness of the random error variance in the products obtained through the EIVD methodology. In future research, incorporating more input datasets within an ECC fusion framework, along with a comprehensive evaluation of the strengths and weaknesses of different products, could lead to developing a more robust transpiration benchmark.

### Potential uncertainty during data processing and evaluation

This study introduces two more potential sources of error. Firstly, data source errors arose from the statistical interpolation utilized for input data. Secondly, errors are associated with our analysis’s partition methods employed as references.

We explain our approach in the Data and Methods sections, which involves downscaling the GLDAS and GLEAM datasets from 0.25° to 0.1° using statistical techniques. Additionally, we upscale PMLv2 from 0.083° to 0.1° to ensure consistent spatial resolution across datasets. However, it is worth noting that our downscaling process does not incorporate supplementary information such as elevation, land cover changes, and other meteorological factors. Consequently, some errors in the statistical downscaling may exist compared to more intricate methodologies^65^. Nevertheless, it is reassuring to highlight that our study yields reliable transpiration estimates characterized by reasonable spatial patterns and consistent trends over multiple years. For future research endeavours, incorporating elevation and other pertinent data into the downscaling and upscaling procedures, possibly through methods like random forests, could offer potential enhancements in accuracy.

The sources of errors in our analysis are also linked to variations stemming from different partitioning methods. In our site-scale assessment of the fused results, we utilize mean transpiration values derived from three sets of ET partitioning methods as our reference benchmark. As outlined in the methodology section, these three methods exhibit some differences, particularly in their assumptions regarding specific conditions for T≈ET. Consequently, relying solely on the results from a single partitioning method as a reference would not have provided a sufficiently reliable basis for our evaluations^53^. Therefore, we chose to use the average values as our reference standard. It is important to note that numerous other ET partitioning methods were available^18^, and in this study, we select three commonly used ones. Utilizing results from alternative methods as references could have led to different conclusions.

### Validation, potential applications, and future enhancements

In this study, our primary focus concentrates on site-scale validation against partitioned results from FLUXNET sites. However, with the continual advancement and increasing availability of sap flow observations, offering a more direct approach for assessing transpiration estimates, the integration of sap flow data holds significant promise for further enhancing our product’s validation and overall quality. Notably, recent work by Bittencourt *et al*.^66^ successfully validated the reliability of GLEAM transpiration products utilizing SAPFLUXNET data^67^. Their study introduces a data-processing approach, enabling SAPFLUXNET data as benchmarks. Nevertheless, it is essential to acknowledge that their findings also emphasized the inherent uncertainty in sap flow data. Therefore, we advocate for a comprehensive comparison between observed and estimated variations, as demonstrated in their study using Z-scores. In summary, we maintain that the site references chosen in this study were relatively robust. However, future investigations may consider exploring alternative reference sources to provide insights into potential disparities when incorporating sap flow data into the analysis.

Turning our attention to potential applications of our product, we propose three key avenues. (i) Global Transpiration Trends: Our product offers insights into current transpiration patterns and enables the examination of multiyear trends in global transpiration. Such long-term trends are essential in understanding how ecosystems respond to changing environmental conditions, especially in the context of a warming climate; (ii) Transpiration-to-Evapotranspiration Ratio: Beyond trends in global transpiration, our product provides another metric—the ratio of transpiration to evapotranspiration. Understanding variations in this ratio can lead to more efficient water resource management strategies and improved predictions of water availability in different regions; (iii) Attribution Analysis: Our product can serve as a valuable tool for attribution analysis, helping researchers identify the drivers behind transpiration patterns. This knowledge is vital for disentangling the roles of climate variability, land-use changes, and other factors in shaping terrestrial water fluxes.

We have outlined a proactive approach to future updates in our ongoing commitment to providing a robust and reliable transpiration product. First and foremost, we will rigorously validate and incorporate more reliable datasets into our fusion process. This validation ensures that the data sources we integrate meet high-quality standards. Furthermore, as the scientific community continually improves and updates input datasets, we are dedicated to promptly adapting our product to accommodate the latest versions. This agility ensures that our transpiration estimates remain up-to-date and reflect the most current scientific understanding.

### Supplementary information

Supplementary

## Data Availability

The present study employed the MATLAB r2020a version (compatible with newer versions) to implement the EIVD algorithm and conducted data fusion. The code (“EIVD_example.m”) has been uploaded along with the data via figshare for public access and download^64^.
