# Supplementary material for: A harmonized global gridded transpiration product based on collocation analysis
Source: Sci Data. 2024 Jun 7;11:604. doi: 10.1038/s41597-024-03425-7 (PMC11161592; doi:10.1038/s41597-024-03425-7)
Supplement: Supplementary file 1 — Supplementary [file 41597_2024_3425_MOESM1_ESM.docx]

**A harmonized global gridded transpiration product based on collocation analysis.**

Changming Li^1^, Juntai Han^1^, Ziwei Liu^1^, Zhuoyi Tu^1^, Hanbo Yang^1*^

^1^State Key Laboratory of Hydroscience and Engineering, Department of Hydraulic Engineering, Tsinghua University. Beijing 100084, China

^*^Correspondence: Hanbo Yang ([yanghanbo@tsinghua.edu.cn](mailto:yanghanbo@tsinghua.edu.cn) )

Here we provide detailed description of the extend collocation (EC) analysis and fusion.

For a quadruple collocation (QC) with the input of $[X,Y,Z,W \mathrm{with}\sigma_{\varepsilon_{X}\varepsilon_{Y}}\neq0]$:

| $\mathbf{y}=\left( \begin{aligned} \sigma_{X}^{2} \\ \sigma_{Y}^{2} \\ \sigma_{Z}^{2} \\ \sigma_{W}^{2} \\ \sigma_{XY} \\ \frac{\sigma_{XZ}\sigma_{XW}}{\sigma_{ZW}} \\ \frac{\sigma_{YZ}\sigma_{YW}}{\sigma_{ZW}} \\ \frac{\sigma_{ZX}\sigma_{ZW}}{\sigma_{XW}} \\ \frac{\sigma_{ZY}\sigma_{ZW}}{\sigma_{YW}} \\ \frac{\sigma_{WX}\sigma_{WZ}}{\sigma_{XZ}} \\ \frac{\sigma_{WY}\sigma_{WZ}}{\sigma_{YZ}} \\ \frac{\sigma_{XW}\sigma_{YZ}}{\sigma_{WZ}} \\ \frac{\sigma_{XZ}\sigma_{YW}}{\sigma_{ZW}} \end{aligned} \right)_{\boldsymbol{13}\mathbf{x1}}\mathbf{A=}\left( \begin{matrix} \mathbf{I}_{\mathbf{5x5}} & \mathbf{I}_{\mathbf{5x5}} \\ \left( \begin{matrix} 1 & 0 & 0 & 0 & 0 \\ 0 & 1 & 0 & 0 & 0 \\ 0 & 0 & 1 & 0 & 0 \\ 0 & 0 & 1 & 0 & 0 \\ 0 & 0 & 0 & 1 & 0 \\ 0 & 0 & 0 & 1 & 0 \\ 0 & 0 & 0 & 0 & 1 \\ 0 & 0 & 0 & 0 & 1 \end{matrix} \right)_{\boldsymbol{8}\mathbf{x5}} & \mathbf{0}_{\boldsymbol{8}\mathbf{x5}} \end{matrix} \right)_{\boldsymbol{13}\mathbf{x10}}\mathbf{x}=\left( \begin{aligned} \beta_{X}^{2}\sigma_{\Theta}^{2} \\ \beta_{Y}^{2}\sigma_{\Theta}^{2} \\ \beta_{Z}^{2}\sigma_{\Theta}^{2} \\ \beta_{W}^{2}\sigma_{\Theta}^{2} \\ \beta_{X}\beta_{Y}\sigma_{\Theta}^{2} \\ \sigma_{\varepsilon_{X}}^{2} \\ \sigma_{\varepsilon_{Y}}^{2} \\ \sigma_{\varepsilon_{Z}}^{2} \\ \sigma_{\varepsilon_{W}}^{2} \\ \sigma_{\varepsilon_{X}\varepsilon_{Y}} \end{aligned} \right)_{\boldsymbol{10}\mathbf{x1}}$ | (1) |
| --- | --- |

Where $\mathbf{I}_{\mathbf{mxn}}$ is identity matrix; $\mathbf{0}_{\mathbf{mxn}}$ is the zero matrix. The least-squared solution for unknown $\mathbf{x}$ is then solved by:

|  | $\mathbf{x=}\left( \mathbf{A}^{\mathbf{T}}\mathbf{A} \right)^{\mathbf{-1}}\mathbf{A}^{\mathbf{T}}\mathbf{y}$ | (2) |
| --- | --- | --- |

The matrix $\left( \mathbf{A}^{\mathbf{T}}\mathbf{A} \right)$ must have full rank (invertible) to guarantee that the collocation system is solvable.

The error data obtained through the EC method can be harnessed to perform weighted averaging of input values. This involves determining the most advantageous blend of forecasts, guided by the mean square error (MSE) criterion ^1^. In this context, "optimal" signifies minimizing the variance of residual random errors through a least squares approach. Expressing this mathematical concept formally, we arrive at the following weighted average representation:

|  | $\overline{x}={\vec{\mathbf{W}}}^{T}\vec{\mathbf{X}}\boldsymbol{=}\sum_{i=1}^{N} \omega_{i}x_{i}$ | (3) |
| --- | --- | --- |

where $\overline{x}$ is the merged estimate; $\vec{\mathbf{X}}\mathbf{=}\left[ x_{1},\ldots,x_{n} \right]^{T}$ contains the temporally collocated estimates from $N$ different parent products, which are merged with relative zero-mean random error $\vec{\boldsymbol{e}}=\left[ \varepsilon_{1},\ldots,\varepsilon_{n} \right]^{T}$; and $\vec{\mathbf{W}}=\left[ \omega_{1},\ldots, \omega_{n} \right]^{T}$ contains the weights assigned to these estimates, where $\omega_{i}\in\left[ 0,1 \right]$ and $\sum\omega_{i}=1$ ensuring an unbiased prediction.

The averaging weights can be expressed as the solution to the problem:

|  | ${\min f\left( \vec{\mathbf{W}} \right)\mathbb{=E}\left( {\vec{\boldsymbol{e}}}^{T}\vec{\mathbf{W}} \right)}^{2}$ | (4) |
| --- | --- | --- |

where $\mathbb{E}$() is the operator for mathematical expectation, the solution of this problem is determined by the individual random error characteristics of the input data sets and can be derived from their covariance matrix ^1–3^:

|  | $\vec{\mathbf{W}}=\left( {\vec{\mathbf{I}}}^{T}\mathbb{E}\left( \vec{\boldsymbol{e}}{\vec{\boldsymbol{e}}}^{T} \right)^{-1}\vec{\mathbf{I}} \right)^{-1}\mathbb{E}\left( \vec{\boldsymbol{e}}{\vec{\boldsymbol{e}}}^{T} \right)^{-1}\vec{\mathbf{I}}$  $\sigma_{\varepsilon_{\overline{x}}}^{2}=\left( {\vec{\mathbf{I}}}^{T}\mathbb{E}\left( \vec{\boldsymbol{e}}{\vec{\boldsymbol{e}}}^{T} \right)^{-1}\vec{\mathbf{I}} \right)^{-1}$ | (5) |
| --- | --- | --- |

where $\mathbb{E}\left( \vec{\boldsymbol{e}}{\vec{\boldsymbol{e}}}^{T} \right)$is the $N\times N$ error covariance matrix that holds the random error variance $\sigma_{\varepsilon_{i}}^{2}$ of the parent products in the diagonals and relative error covariances $\sigma_{\varepsilon_{i}\varepsilon_{j}}$ in the off-diagonals; $\vec{\mathbf{I}}=\left[ 1,\ldots,1 \right]^{T}$ is an ones-vector of length $N$; and $\sigma_{\varepsilon_{\overline{x}}}^{2}$ is the resulting random error variances of the merged estimate.

In this case, we can identify four sets of products as inputs ($N=4$). In this scenario, we consider the possibility of error homogeneity, assuming a non-zero ECC exists between inputs 1 and 2. Thus, the error matrix can be represented as:

|  | $\mathbb{E}\left( \vec{\boldsymbol{e}}{\vec{\boldsymbol{e}}}^{T} \right)=\left[ \begin{matrix} \sigma_{\varepsilon_{1}}^{2} & \sigma_{\varepsilon_{1}\varepsilon_{2}} & 0 & 0 \\ \sigma_{\varepsilon_{1}\varepsilon_{2}} & \sigma_{\varepsilon_{2}}^{2} & 0 & 0 \\ 0 & 0 & \sigma_{\varepsilon_{3}}^{2} & 0 \\ 0 & 0 & 0 & \sigma_{\varepsilon_{4}}^{2} \end{matrix} \right]$ | (6) |
| --- | --- | --- |

The weights can then be written as:

|  | $\vec{\mathbf{W}}=\left\{ \begin{aligned} \frac{\sigma_{\varepsilon_{2}}^{2}-\sigma_{\varepsilon_{1}\varepsilon_{2}}}{\left( \sigma_{\varepsilon_{1}}^{2}\sigma_{\varepsilon_{2}}^{2}-\sigma_{\varepsilon_{1}\varepsilon_{2}}^{2} \right)\mathbb{*z}} \\ \frac{\sigma_{\varepsilon_{1}}^{2}-\sigma_{\varepsilon_{1}\varepsilon_{2}}}{\left( \sigma_{\varepsilon_{1}}^{2}\sigma_{\varepsilon_{2}}^{2}-\sigma_{\varepsilon_{1}\varepsilon_{2}}^{2} \right)\mathbb{*z}} \\ \frac{1}{\sigma_{\varepsilon_{3}}^{2}\mathbb{*z}} \\ \frac{1}{\sigma_{\varepsilon_{4}}^{2}\mathbb{*z}} \end{aligned} \right.$  $\mathbb{z}=\frac{\sigma_{\varepsilon_{1}}^{2}+\sigma_{\varepsilon_{2}}^{2}-2\sigma_{\varepsilon_{1}\varepsilon_{2}}}{\sigma_{\varepsilon_{1}}^{2}\sigma_{\varepsilon_{2}}^{2}-\sigma_{\varepsilon_{1}\varepsilon_{2}}^{2}}+\frac{1}{\sigma_{\varepsilon_{3}}^{2}}+\frac{1}{\sigma_{\varepsilon_{4}}^{2}}$ | (7) |
| --- | --- | --- |

It is essential to acknowledge that before applying these weights for merging the data sets, it is necessary to address any existing systematic differences. Typically, this is achieved by rescaling the data sets to a standardized data space.

References

1. Bates, J. M. & Granger, C. W. The combination of forecasts. *Journal of the Operational Research Society* **20**, 451–468 (1969).

2. Gruber, A., Dorigo, W. A., Crow, W. & Wagner, W. Triple Collocation-Based Merging of Satellite Soil Moisture Retrievals. *IEEE Trans. Geosci. Remote Sensing* **55**, 6780–6792 (2017).

3. Kim, S., Sharma, A., Liu, Y. Y. & Young, S. I. Rethinking satellite data merging: from averaging to SNR optimization. *IEEE Transactions on Geoscience and Remote Sensing* **60**, 1–15 (2021).
